# ﻿Checklist of hosts, illustrated geographical range, and ecology of tick species from the genus *Ixodes* (Acari, Ixodidae) in Russia and other post-Soviet countries

**DOI:** 10.3897/zookeys.1201.115467

**Published:** 2024-05-14

**Authors:** Denis Fedorov, Sándor Hornok

**Affiliations:** 1 HUN-REN-UVMB Climate Change: New Blood-sucking Parasites and Vector-borne Pathogens Research Group, Budapest, Hungary HUN-REN-UVMB Climate Change: New Blood-sucking Parasites and Vector-borne Pathogens Research Group Budapest Hungary; 2 Zoological Institute of the Russian Academy of Sciences (ZIN-RAS), St. Petersburg, Russia Zoological Institute of the Russian Academy of Sciences (ZIN-RAS) St. Petersburg Russia; 3 Department of Parasitology and Zoology, University of Veterinary Medicine, Budapest, Hungary University of Veterinary Medicine Budapest Hungary

**Keywords:** Acari, Aves, Ixodidae, Mammalia, Reptilia, subgenus, taxonomy

## Abstract

Hard ticks (Acari: Ixodidae) are the economically and ecologically most important blood-sucking arthropod vectors that can transmit disease agents under temperate climate. In this group, the highest number of species (currently nearing 270) belongs to the genus *Ixodes*. For this review, more than 400 papers related to this genus in the context of Russia were checked for data on the host records, locations of collection, as well as ecology of assigned tick species. This monograph compensates for the lack of a similarly comprehensive English-language overview of *Ixodes* species in the region of Russia for nearly half century, and also makes a large set of data easily available for international readers, which is especially important if the original source is difficult to access from outside this country. In addition, the data from a significant number of papers on this topic available only in the Russian language are made accessible through this work.

## ﻿Introduction

Russia is the largest country of the globe, covering nearly one third of the territory of Eurasia and 1/8^th^ of the entire Earth’s landmass. It belongs to the Palearctic Zoogeographic Region (Guglielmone et al. 2023). The ecosystems of Russia are very diverse, including polar deserts, tundra, forest tundra, taiga, mixed and broad-leaved forests, forest steppe, steppe, semi-desert, and subtropics. At least 1100 species of terrestrial vertebrates are known to occur in this country, of which 65% of the territory is considered virtually untouched by economic and other human activities (CBD 2023).

With such a vast area, the broad spectrum of suitable habitats and vertebrate hosts in the background, the tick fauna of Russia was extensively studied. Although there was an enormous collection of data published in English (Anastos 1957), because it is more than half a century old, it is outdated. Moreover, the most well-known source describing the taxonomic diversity of Ixodidae Koch in this country and its nearby regions was compiled decades ago (Filippova 1977; 1997), and is only available in the Russian language. This book on ixodid species in Russia included 34 *Ixodes* Latreille species, which is updated to 37 by adding species with more recent data, as exemplified by *Ixodesprokopjevi* Emelyanova and *I.ghilarovi* Filippova & Panova, as well as *I.turdus* Nakatsudi with its first and single record (Bolotin and Kolonin 1979). Recent work has also been published including a list of hard tick species known to be indigenous in Russia (Guglielmone et al. 2023) with indications of tick species of other post-Soviet countries. However, the latter does not consider their specific locations or host records and various distinctive features of biology relevant to certain regions. Less studied species (some often known exclusively from these territories by single or a limited number of findings) are also reviewed here in more detail, in particular with the addition of precise data on their type specimens.

The need was recognized for a comprehensive work that would contain data and references from the last decades, written in English, which would thus be accessible by experts and anyone interested in the current ixodid fauna and its supportive hosts in the vast geographical and biotope range of Russia, as well as several other post-Soviet territories. In this review the authors tried to compensate for this scarcity of fresh information on hard ticks occurring in Russia and former states of the Soviet Union, targeting the most species-rich genus, *Ixodes*. Although the checklist and georeferenced data might still contain gaps, this work is also intended to be used as baseline data for the unfolding quest to discover and to describe not-yet-known ixodid species in this extensive geographical range.

## ﻿Materials and methods

The relevance of publications used in this review was searched in databases using the keywords of *Ixodes* species, their hosts, and locality or region. The following databases were used: Library of the Russian Academy of Sciences (including its department at the Zoological Institute of the Russian Academy of Sciences), Springer Link, Web of Science, Zoological Record, Google Scholar, and CyberLeninka – the Russian scientific electronic library. However, a limited number of works was excluded from consideration and inclusion in the review due to the absence of scientific background and/or indeterminate data. Similarly, papers with repetitive data (i.e., not adding new tick-host associations, geographical locations to existing literature data) are not cited.

The same databases were used for searching and estimating the data on *Ixodes* from the post-Soviet territories, reviewed in this checklist: Russia, Belarus, Ukraine, Moldova, Georgia, Azerbaijan, Armenia, Kazakhstan, Kyrgyzstan, Uzbekistan, Turkmenistan, and Tajikistan. The Baltic states (Estonia, Latvia, and Lithuania) were excluded from the consideration due to the availability of recently updated tick checklists, as well as the well-studied tick fauna (Paulauskas et al. 2010; Kitrytė and Baltrūnaitė 2023), which is also very similar to the tick fauna of neighboring Belarus and northwestern Russia.

Within Prostriata (genus *Ixodes*), tick species names are arranged according to their subgenera and are used sensu Guglielmone et al. (2014). The Latin names of tick species are written according to Guglielmone et al. (2023). The only exception is *Ixodesfilippovae* Černý which we consider a synonym of *Ixodescrenulatus* Koch according to Filippova (1958a, 1977). The names of host species are written in accordance with their international English names, as well as the current Latin names using the online databases, such as ASM Mammal Diversity Database (https://www.mammaldiversity.org/index.html) as well as Avibase (https://avibase.bsc-eoc.org/) and Reptile Database (http://www.reptile-database.org/).

## ﻿Systematics

### ﻿Class Arachnida

**Order Ixodida**

**Family Ixodidae Koch, 1844**

**Genus *Ixodes* Latreille, 1795**

#### Subgenus ﻿Ceratixodes Neumann, 1902: 115

##### ﻿ Ixodes uriae

Taxon classificationAnimaliaIxodidaIxodidae

White, 1852

90261B82-C4E7-55FE-A74D-B3B25BAC2593

Ixodes uriae White, 1852: 208.
Ixodes jacksoni Hoogstraal, 1967: 37.
Ixodes fimbriatus Kramer & Neumann, 1883: 527; Neumann 1911: 29.
Ixodes borealis Kramer & Neumann, 1883: 526; Neumann 1911: 29.
Ixodes hirsutus Birula, 1895: 353; Arthur 1963: 152.
Ixodes putus (Pickard-Cambridge, 1876): 260; Neumann 1899: 125; Schulze 1938: 12.
Ixodes putus procellariae Schulze, 1930: 123; Zumpt 1952: 12.

###### Recorded hosts.

**Aves**: auks - birds of the family Alcidae, namely: *Alcatorda* Linnaeus (razorbill), *Cepphusgrylle* Linnaeus (black guillemot), *Fraterculaarctica* Linnaeus (common puffin), *Uriaaalge* (Pontoppidan) (common guillemot), *Urialomvia* Linnaeus (Brünnich’s guillemot) (Filippova 1977).

Occasional hosts include *Fraterculacirrhata* (Pallas) (tufted puffin) and also various species of gulls and kittiwakes (Laridae): *Rissabrevirostris* (Bruch) (red-legged kittiwake), *R.tridactyla* (Linnaeus) (black-legged kittiwake) (Karpovich 1970; Dietrich et al. 2012), as well as fulmars (Procellariidae) – *Fulmarusglacialis* Linnaeus (northern fulmar) and cormorants (Phalacrocoracidae) – *Phalacrocoraxcapillatus* (Temminck and Schlegel) (Japanese cormorant), *Urilepelagicus* (Pallas) (pelagic cormorant), *Urileurile* (Gmelin) (red-faced cormorant) (Lvov et al. 1972b, 1975; Filippova 1977; Dietrich et al. 2012; Duron et al. 2014). A single atypical case of parasitism on *Motacillaalba* Linnaeus (white wagtail) (Motacillidae) was also reported (Karpovich 1970).

###### Recorded locations

**Figure 1.**
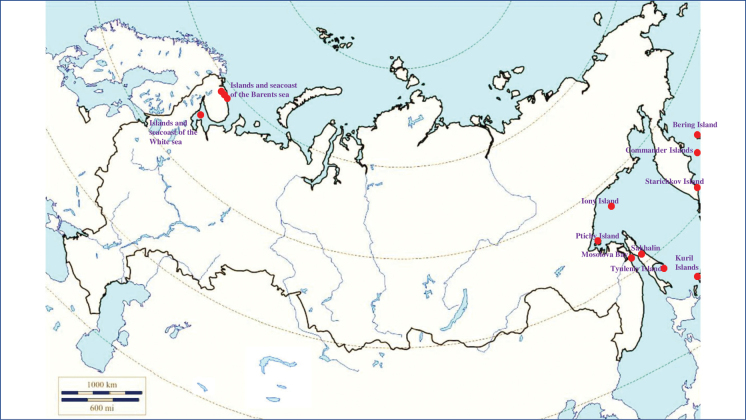
Map of Russia and neighboring countries showing the locations where *Ixodesuriae* was reported.

**(Fig. 1). Murmansk seacoast (Russia)**: islands and seashores of the White Sea and also the Barents Sea (Karpovich 1971), namely: the Kuvshin Island and Kharlov Island (Karpovich 1970), Podpakhta Bay (Bekleshova et al. 1970), Dvorovaya Bay (Flint and Kostryko 1967), Seven Islands Reserve (Belopolskaya 1952; Karpovich 1973). **The Far East (Russia)**: Mosolova Bay (the northern coast of the Strait of Tartary) (Savitskaya 1975), Bering Island (Lvov et al. 1975; Dietrich et al. 2012), Ptichy Island and Starichkov Island (Dietrich et al. 2012), Iony Island (Lvov et al. 1975), Kuril Islands (Lvov et al. 1975; Dietrich et al. 2012), Tyuleniy Island (Karpovich 1971; Lvov et al. 1972a; Filippova 1977), Sakhalin (Lvov et al. 1972a), Commander Islands (Dietrich et al. 2012).

###### Ecology and other information.

*Ixodesuriae* is the only representative of the subgenus ﻿Ceratixodes in the tick fauna of Russia and the northern hemisphere in general. As a nidicolous parasite of seabirds living in colonies, it is a species with a circumpolar distribution, occurring on oceanic coasts and islands of both the northern and southern hemispheres, from the polar regions to the subtropical zone (Wilson 1967; Filippova 1977).

In the northern hemisphere, this tick species is strongly associated with seabirds of the family Alcidae. The high degree of nest conservativity of these birds contributes to supporting a considerable number of ticks in bird colonies, which use the same places for many years (Karpovich 1971). The occasional hosts of *I.uriae* usually become involved in its life cycle in mixed bird colonies, where nests of typical and atypical hosts are located very close to each other (Violovich 1962b). In absence of auks, it may also use, for example, cormorants as exclusive hosts, as reported on the Kuril Islands (Lvov et al. 1975; Dietrich et al. 2012). In the southern hemisphere it was noted that penguins (Spheniscidae) are more typical hosts; this can be explained by similarities in the habits of these birds to those of puffins and guillemots.

There were noted rare records of adults from Carnivora: Mustelidae, and nymphs from Rodentia: Muridae (Eley 1977; Jaenson and Jensen 2007; Baggs et al. 2011; Guglielmone et al. 2020) and even humans (Karpovich 1971; Keirans and Lacombe 1998; Martyn 1998; Smith et al. 2006; Jaenson and Jensen 2007).

#### ﻿Subgenus ﻿Eschatocephalus Frauenfeld, 1853: 55

##### ﻿ Ixodes simplex

Taxon classificationAnimaliaIxodidaIxodidae

Neumann, 1906

5AE9E640-7719-587E-8497-F8BCF49991A9

Ixodes simplex Neumann, 1906: 197.
Ixodes audyi Kohls, 1955: 1; Clifford et al. 1973: 489.
Ixodes spiculae Arthur, 1956: 180.
Ixodes pospelovae Emchuk, 1955: 606; Beaucournu 1966: 495.
Ixodes chiropterorum Babos & Janisch, 1958: 389; Beaucournu 1966: 495.

###### Recorded hosts.

**Mammalia**: *Myotisblythii* Tomes (lesser mouse-eared bat), *Miniopterusschreibersii* (Kuhl) (common bent-wing bat), *Nyctalusleisleri* (Kuhl) (lesser noctule) (Filippova 1977).

###### Recorded locations

**Figure 2.**
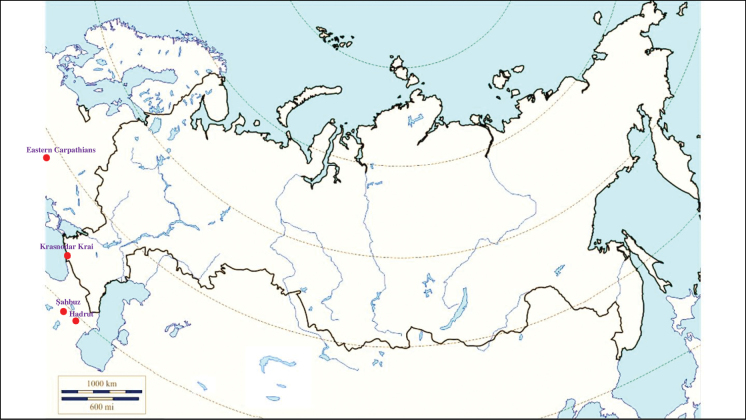
Map of Russia and neighboring countries showing the locations where *Ixodessimplex* was reported.

**(Fig. 2). Russia**: Krasnodar Krai – outskirts of Sochi (Emchuk 1955; Filippova 1972). **Ukraine**: eastern Carpathians – outskirts of Solotvyn and Rakhiv (Emchuk 1955; Filippova 1972). **Azerbaijan**: outskirts of Şahbuz and Hadrut (Emchuk 1955; Filippova 1972).

###### Ecology and other information.

*Ixodessimplex* is a tick species specialized for bats as hosts (Filippova 1977). This species is mainly monoxenous and can be found usually on the common bent-wing bat although some other species of the Chiroptera may also act as hosts, especially which share colonies with its main host (Beaucournu 1967). Some rare cases of human infestation are also recorded (Okino et al. 2010; Péter et al. 2021).

##### ﻿ Ixodes vespertilionis

Taxon classificationAnimaliaIxodidaIxodidae

Koch, 1844

334033B4-AC8D-5E16-BE30-9E8283A48497

Ixodes vespertilionis Koch, 1844b: 232.
Ixodes longipes Lucas: Neumann 1901: 249.
Ixodes pagurus Neumann, 1911: 28.
Ixodes nodulipes (Kolenati): Neumann 1911: 28.
Ixodes troglodytes Schmidt in Frauenfeld: Neumann 1901: 249.
Eschatocephalus gracilipes Frauenfeld: Estrada-Peña 1989: 165.
Eschatocephalus nodulipes Santos Dias: Santos Dias 1961: 229.
Eschatocephalus seidlitzii Koch: Neumann 1911: 30.
Eschatocephalus frauenfeldi Koch: Neumann 1901: 249.
Eschatocephalus seidlitzi Koch: Neumann 1901: 249.
Eschatocephalus vespertilionis (Koch): Neumann 1901: 249.
Eschatocephalus exaratus (Kolenati): Neumann 1901: Santos Dias 1961: 229.
Eschatocephalus flavipes (Koch): Doss and Anastos 1977: 34.

###### Recorded hosts.

**Mammalia**: *Eptesicusserotinus* (Schreber) (serotine bat), *Myotisblythii* (lesser mouse-eared bat) (Filippova 1977), pond bat *Myotisdasycneme* (Boie) (Starikov et al. 2017b), Daubenton’s bat *Myotisdaubentonii* (Kuhl) (Orlova et al. 2011), *Myotismyotis* (Borkhausen) (greater mouse-eared bat) (Filippova 1977), *Myotismystacinus* (Kuhl) (whiskered bat) (Bobkova 2003), *Nyctalusnoctula* (Schreber) (common noctule), *Pipistrelluspipistrellus* (Schreber) (common pipistrelle), *Rhinolophusferrumequinum* (Schreber) (greater horseshoe bat), *Rhinolophushipposideros* (Bechstein) (lesser horseshoe bat), *Rhinolophusmehelyi* Matschie (Mehely’s horseshoe bat) (Filippova 1977).

###### Recorded locations

**Figure 3.**
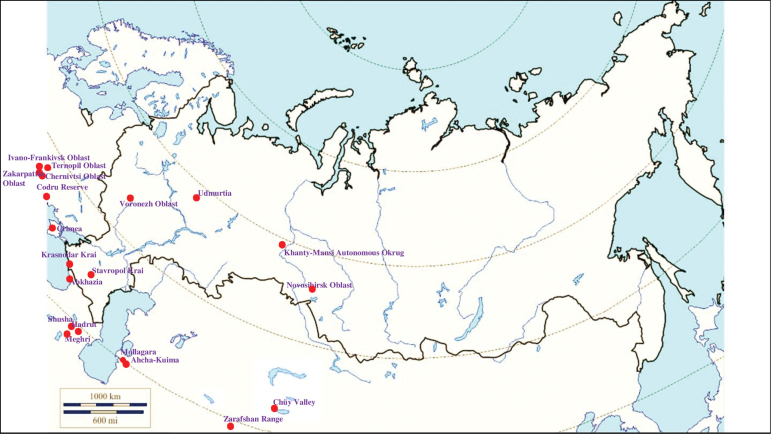
Map of Russia and neighboring countries showing the locations where *Ixodesvespertilionis* was reported.

**(Fig. 3). Russia**: Udmurtia (Orlova et al. 2011), Voronezh Oblast (Usmsnskyi pine forest), (Khitsova and Sherstyanykh 2014), Novosibirsk Oblast (resort Lake Karachi) (Fedorov 2016), Khanty-Mansi Autonomous Okrug (outskirts of the urban locality Mortka) (Starikov et al. 2017b), Krasnodar Krai (Sochi National Park) (Romashin 2021), Stavropol Krai (Tsapko 2019). **Ukraine**: Ivano-Frankivsk Oblast, Chernivtsi Oblast, Ternopil Oblast, Zakarpattia Oblast, Crimea (Bobkova 2003). **Moldova**: Codru Reserve (Dniester-Prut interfluve) (Uspenskaya 1987). **Georgia**: Abkhazia (Kerbabaev 2011). **Azerbaijan**: Shusha, Hadrut (Filippova 1972). **Armenia**: Meghri (Ogandzhanyan 1949). **Kyrgyzstan**: Chüy Valley (Fedorova 2012a). Turkmenistan: rural localities Ahcha-Kuima and Mollagara (Dubinin and Bregetova 1952). **Tajikistan**: northern spurs of the Zarafshan Range (Filippova 1972).

###### Ecology and other information.

*Ixodesvespertilionis* Koch is a species of ixodid ticks associated with bats as typical hosts (Filippova 1977), mostly from the families Rhinolophidae and Vespertilionidae. Usually, *I.vespertilionis* can be found in caves inhabited by bats. Occasional findings in Central Russia and Siberia are considered to result from accidental transportation.

#### Subgenus ﻿﻿Filippoviella Apanaskevich, Greiman & Fedorov, 2024: 229

##### ﻿ Ixodes ghilarovi

Taxon classificationAnimaliaIxodidaIxodidae

Filippova & Panova, 1988

90F985E3-8870-5750-A768-BA3F210F7A50

Ixodes ghilarovi Filippova & Panova, 1988: 212.

###### Recorded hosts.

**Mammalia**: *Apodemusflavicollis* Melchior (yellow-necked field mouse), *Chionomysgud* Satunin (Caucasian snow vole), *Chionomysnivalis* (Martins) (European snow vole), *Microtusdaghestanicus* (Shidlovsky) (Daghestan pine vole), *Nothocricetulusmigratorius* (Pallas) (grey dwarf hamster), *Sorexraddei* Satunin (Radde’s shrew) (Filippova and Panova 1989; Filippova and Stekol’nikov 2007).

###### Recorded locations

**Figure 4.**
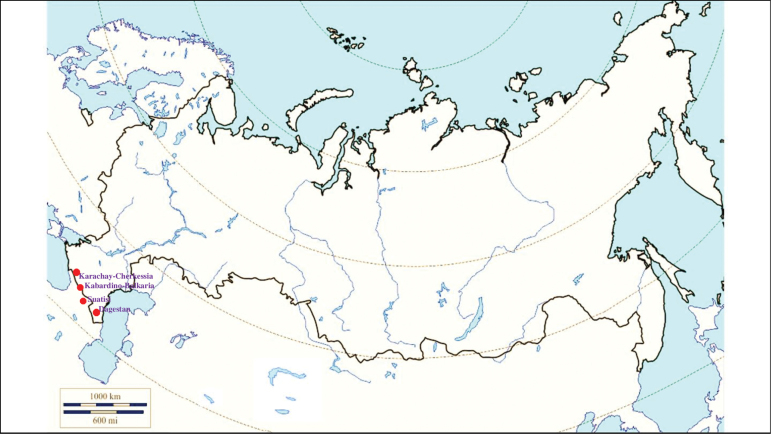
Map of Russia and neighboring countries showing the locations where *Ixodesghilarovi* was reported.

**(Fig. 4). Russia**: Dagestan – the valley of the Akhtychay River which is the right tributary of the Samur River near the confluence of these rivers, ~ 1000 m a.s.l. and at the same location near rural locality Khnov, ~ 1700 m a.s.l.; the valley of the Avar Koysu River, ~ 1000 m a.s.l. (Filippova and Panova 1989); Kabardino-Balkaria, Bezengi gorge – 1550–2500 m a.s.l. and Karachay-Cherkessia – 1900–2200 m a.s.l. (Filippova and Stekol’nikov 2007). **Georgia**: Mtskheta-Mtianeti region, Kazbegi Municipality, outskirts of the hamlet Suatisi, 2200 m a.s.l. (Filippova and Panova 1989).

###### Ecology and other information.

*Ixodesghilarovi* is the second representative of the subgenus ﻿Filippoviella in the Palearctic tick fauna together with *I.trianguliceps* but known at the current moment exclusively from several locations of the Caucasus (Filippova and Panova 1988). The species was found only in rocky biotopes on the slopes containing xerophilous herbaceous-shrub vegetation consisting of many endemics of Southern Dagestan (Filippova and Panova 1989).

Further investigations of this poorly studied tick species are of undoubted interest. *Ixodesghilarovi* has certain common structural features with the African species *I.alluaudi*, for example the presence of auriculae, especially visible in nymphs of both species (Filippova 2010); molecular analysis is also necessary to obtain more data on interspecific connections of these ticks and inside the subgenus in general. The host-parasite relations of *I.ghilarovi* and its distribution and habitats are probably wider than it is known today. The seasonality of *I.ghilarovi* and its role as a vector of tick-borne infections remain unknown.

The type specimens of *I.ghilarovi* are deposited at the Zoological Institute of the Russian Academy of Sciences and include the holotype: nymph; Russia, 25, Daghestan, Samur Mt. Range, near Akhty Village, River Akhtychay valley, ~ 1000 m a. s. l., *Chionomysgud*, Sat., 24.5.1980, coll. I.V. Panova; FBM 610a, 610b and the paratypes: 4 nymphs; FBM I610a, I610b. Description – Filippova and Panova 1989: 419–421 (female, larva; male unknown) (Filippova 2008).

##### ﻿ Ixodes trianguliceps

Taxon classificationAnimaliaIxodidaIxodidae

Birula, 1895

C18A98D9-620F-5523-BA25-931769A9F33D

Ixodes trianguliceps Birula, 1895: 358.
Ixodes nivalis Rondelli, 1928: 85; Pomerantsev 1950: 84.
Ixodes tenuirostris Neumann, 1901: 286.
Endopalpiger heroldi Schulze, 1939: 35; Černý 1959: 156.

###### Recorded hosts.

**Mammalia**: *Alexandromysoeconomus* (Pallas) (tundra vole), *Apodemusagrarius* (Pallas) (striped field mouse) (dominates as the host in the Udel’ny forest park in St. Petersburg, according to Tretyakov (2009), *Apodemusflavicollis* (yellow-necked field mouse), *Apodemussylvaticus* (Linnaeus) (wood mouse), *Apodemusuralensis* Pallas (Ural field mouse), *Arvicolaamphibius* (Linnaeus) (European water vole), *Chionomysgud* (Caucasian snow vole), *Chionomysnivalis* (European snow vole), *Craseomysrufocanus* (Sundevall) (grey red-backed vole), *Cricetuscricetus* (Linnaeus) (European hamster), *Crociduraleucodon* (Hermann) (bicolored shrew), *Crocidurasuaveolens* (Pallas) (lesser white-toothed shrew), *Eutamiassibiricus* (Laxmann) (Siberian chipmunk), *Lasiopodomysgregalis* (Pallas) (narrow-headed vole), *Lepuseuropaeus* Pallas (European hare), *Lepustimidus* Linnaeus (mountain hare), *Micromysminutus* (Pallas) (harvest mouse), *Microtusagrestris* (Linnaeus) (short-tailed field vole), *Microtusarvalis* (Pallas) (common vole), *Microtusmajori* (Thomas) (Major’s pine vole), *Microtussocialis* (Pallas) (social vole), *Microtussubterraneus* (de Selys-Longchamps) (European pine vole), *Musmusculus* Linnaeus (house mouse), *Mustelanivalis* Linnaeus (least weasel), *Myodesglareolus* (Schreber) (bank vole), *Myodesrutilus* (Pallas) (northern red-backed vole), *Myopusschisticolor* (Lilljeborg) (wood lemming), *Neomysanomalus* Cabrera (Mediterranean water shrew) (Filippova 1977), *Neomysfodiens* (Pennant) (Eurasian water shrew) (Lutta 1968), *Nyctalusnoctula* (common noctule), *Ochotonaalpina* (Pallas) (alpine pika), *Prometheomysschaposchnikowi* Satunin (long-clawed mole vole), *Rattusnorvegicus* (Berkenhout) (brown rat), *Sciurusvulgaris* Linnaeus (red squirrel), *Sicistabetulina* Pallas (northern birch mouse), *Sorexaraneus* Linnaeus (common shrew), *Sorexcaecutiens* Laxmann (Laxmann’s shrew), *Sorexdaphaenodon* Thomas (Siberian large-toothed shrew) (Filippova 1977), *Sorexisodon* Turov (taiga shrew) (Sapegina 1980), *Sorexminutus* Linnaeus (Eurasian pygmy shrew), *Sorexminutissimus* Zimmermann (Eurasian least shrew) (Filippova 1977), *Sorexroboratus* Hollister (flat-skulled shrew) (Shtilmark 1963; Sapegina 1980), *Spermophilussuslicus* (Güldenstädt) (speckled ground squirrel), *Vulpesvulpes* (Linnaeus) (red fox) (Filippova 1977).

**Aves**: *Anthustrivialis* (Linnaeus) (tree pipit), *Cardueliscarduelis* (Linnaeus) (European goldfinch), *Dendrocoposmajor* (Linnaeus) (great spotted woodpecker), *Emberizacitronella* Linnaeus (yellowhammer), *Nucifragacaryocatactes* (Linnaeus) (Eurasian nutcracker), *Strixuralensis* Pallas (Ural owl), *Turdusviscivorus* Linnaeus (mistle thrush) (Filippova 1977).

**Reptilia**: *Zootocavivipara* (viviparous lizard) (Lichtenstein) (Filippova 1977).

###### Recorded locations

**Figure 5.**
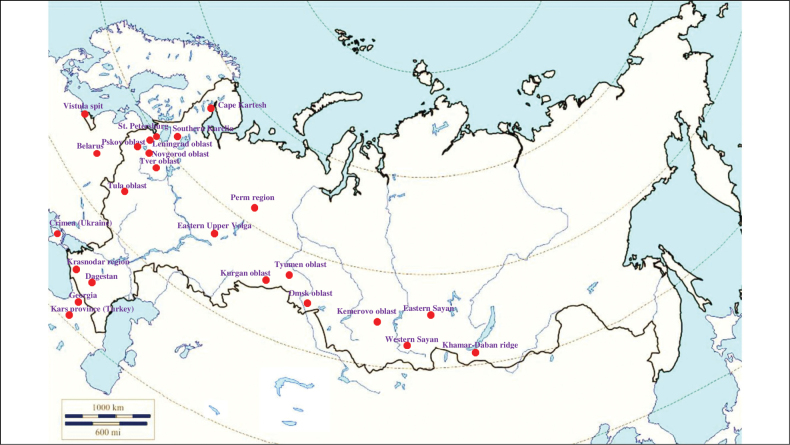
Map of Russia and neighboring countries showing the locations where *Ixodestrianguliceps* was reported.

**(Fig. 5). Russia**: North Karelia – Cape Kartesh (Stanyukovich and Fedorov 2022); Karelia (Lutta 1968) including the village Malaya Gomselga (southern Karelia) (Bespyatova and Bugmyrin 2015; Bespyatova et al. 2019), St. Petersburg (Tretyakov 2009), Leningrad Oblast (Sukhomlinova 1977), Novgorod Oblast (Grigoryeva and Tretyakov 1998), Pskov Oblast – the village Gogolevo (own data, unpublished), Kaliningrad Oblast, the Vistula Spit (own data, unpublished); Tver Oblast (Schipanov and Makhanko 2018), Tula Oblast (Kozlova et al. 2014), Perm Oblast (Korenberg et al. 2015), Eastern Upper Volga (Egorov et al. 2016), Krasnodar Krai and the Caucasus (Shatas 1957; Filippova and Stekol’nikov 2007), Kurgan Oblast (Starikov and Starikova 2021), Tyumen Oblast (Bragina et al. 2013), Omsk Oblast (Rar et al. 2014, 2020), Kemerovo Oblast (Kovalevsky et al. 2018), Western Sayan (Shtilmark 1963), Eastern Sayan (Schluger 1961), Khamar-Daban ridge (Vershinina 1988). **Belarus** (Arzamasov 1963). **Ukraine**: Crimea (Filippova 2010), Polesia (Podobivskyi and Fedonyuk 2017). **Moldova**: north and central Moldova (Uspenskaya et al. 2006). **Georgia**: the village Bakuriani and the Roki Tunnel (Djaparidze 1960). **Armenia**: the whole territory (Ogandzhanyan 1960). **Azerbaijan**: the south of the country (Ogandzhanyan 1960).

###### Ecology and other information.

*Ixodestrianguliceps* Birula has a wide geographical distribution in the Palaearctic region, occurring from the coast of Lake Baikal to Western Europe (Filippova 2010; Estrada-Peña et al. 2018). In the north it reaches northern Karelia and the Scandinavian Peninsula (Fedorov and Leonovich 2021). Also, an isolated southern population of this species was found in the Crimean Peninsula (Filippova 2010) although in other parts of Ukraine it is present in forest zones, such as Polesia (Podobivskyi and Fedonyuk 2017).

The population that was supposed to be isolated in the mountain systems of the Caucasus (Filippova 2010) now seems to be more expanded, as proved by the recent finding in Turkey (Bolu and Kars province, the north of Turkey) (Keskin and Selçuk 2021). The Kars province is located near the border with Georgia, where this species was known before (Djaparidze 1960) and, therefore, the ticks reported from there are probably part of the same Caucasian population.

The map of findings of this tick species in Russia clearly illustrates that it lives in a broad range of forest biotopes throughout a vast territory including the zonal and mountain deciduous and mixed forest of the European type and forests of southern and middle-taiga types. Along the southern border of the largest part of the range in Russia, *I.trianguliceps* occurs in the forest-steppe zone, populating shrubby and forested biotopes. This distinctly correlates with the main habitats of shrews and rodents, because the presence of these small mammals together with well-developed soil litter, plays an important role in the abundance of ticks in the landscape, as it is known that shrews of the genus *Sorex* are the most preferable host for larvae (Randolph 1975).

Interestingly, *I.triangulipeps* was also reported from two bat species (*Myotismyotis* in Poland (Siuda et al. 2009) and *Nyctalusnoctula* in Russia, as well as several bird species and one reptile species (Filippova 1977). These animals are non-typical and occasional hosts for this tick species. The single cases of parasitism on these host species can be a clear indication that *I.trianguliceps* is predominantly an exophilic species, because it is unlikely that ticks could contact bats and birds in a burrow. Findings of this tick species in micropores of burrow tunnels in Belarus in winter (Arzamasov 1963) demonstrate only the ability of its larvae to remain active even during winter.

Phylogenetic trees inferred from the concatenated nucleotide sequences of 10 protein-coding genes of the mitochondrial genome of *I.trianguliceps*, together with consideration of its morphology, justified to establish the new subgenus ﻿Filippoviella and include there *I.trianguliceps* together with aforementioned *I.ghilarovi* (Apanaskevich et al. 2024) both of which used to belong to the subgenus ﻿Exopalpiger.

#### Subgenus ﻿﻿Ixodes Latreille, 1795: 179

##### ﻿ Ixodes apronophorus

Taxon classificationAnimaliaIxodidaIxodidae

Schulze, 1924

F06D4EDC-7835-5A5D-A0F9-10BB8BD3EE87

Ixodes apronophorus Schulze, 1924: 281.
Ixodes arvicolae Warburton, 1926: 55; Morel and Pérez 1978: 201.
Ixodes arvalis Karpov & Popov, 1944: 75; Morel and Pérez 1978: 201.
Ixodes dorrien-smilhi Turk: Morel and Pérez 1978: 201.
Ixodes dorriensmithi Turk: Morel and Pérez 1978: 201.

###### Recorded hosts.

**Mammalia**: *Alexandromysoeconomus* (tundra vole), *Apodemusagrarius* (striped field mouse), *Apodemusflavicollis* (yellow-necked field mouse), *Apodemussylvaticus* (wood mouse), *Arvicolaamphibius* (European water vole), *Cricetuscricetus* (European hamster), *Craseomysrufocanus* (grey red-backed vole), *Erinaceuseuropaeus* Linnaeus (European hedgehog), *Eutamiassibiricus* (Siberian chipmunk), *Lasiopodomysgregalis* (narrow-headed vole), *Lepustimidus* (mountain hare), *Micromysminutus* (Eurasian harvest mouse), *Microtusarvalis* (common vole), *Microtusagrestis* (Linnaeus) (short-tailed field vole), *Musmusculus* (house mouse), *Mustelanivalis* (least weasel), *Mustelasibirica* Pallas (Siberian weasel), *Myodesglareolus* (bank vole), *Myodesrutilus* (northern red-backed vole), *Myopusschisticolor* (wood lemming), *Neomysfodiens* (Eurasian water shrew), *Nothocricetulusmigratorius* (grey dwarf hamster), *Ondatrazibethicus* (Linnaeus) (muskrat), *Rattusrattus* (Linnaeus) (black rat), *Sicistabetulina* (northern birch mouse), *Sorexaraneus* (common shrew), *Sorexcaecutiens* (Laxmann’s shrew), *Sorexdaphaenodon* (Siberian large-toothed shrew), *Sorexisodon* (taiga shrew), *Sorexminutus* (Eurasian pygmy shrew), *Sorexroboratus* (flat-skulled shrew), *Talpaeuropaea* Linnaeus (European mole), *Vulpesvulpes* (red fox) (Filippova 1977).

**Aves**: *Anascrecca* Linnaeus (Eurasian teal) (Adamovich 1968), *Gallinulachloropus* (Linnaeus) (common moorhen) (Filippova 1977), *Motacillaalba* (white wagtail), *Turdusmerula* Linnaeus (common blackbird) (Adamovich 1968).

###### Recorded locations

**Figure 6.**
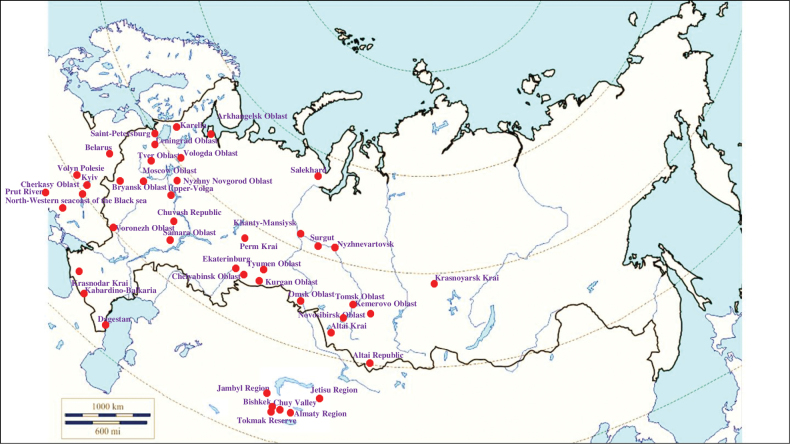
Map of Russia and neighboring countries showing the locations where *Ixodesapronophorus* was reported.

**(Fig. 6). Russia**: Arkhangelsk Oblast (Olenev 1931a), Karelia (Lutta 1976), Saint-Petersburg (Tretyakov 2009), Leningrad Oblast (Sukhomlinova 1977), Vologda Oblast, Tver Oblast (Filippova 1977; Belova et al. 2008), Moscow Oblast (Mosolov 1961), the whole territory of the Upper-Volga (Egorov et al. 2016), Samara Oblast (Kirillova and Kirillov 2008a), Bryansk Oblast (Adamovich 1968), Voronezh Oblast, Nyzhny Novgorod Oblast (Solovyov 1966), Chuvash Republic (Petrov et al. 1967), Krasnodar Krai (Kalita and Pelipeychenko 1957; Shevchenko et al. 1960), Kabardino-Balkaria (Bittirova et al. 2019), Dagestan (Aliev et al. 2012), Perm Krai, Chelyabinsk Oblast (Filippova 1958a), Ekaterinburg (Chernousova and Tolkachyov 2009), Omsk Oblast (Znamenskiy district and Bolsheukov district) (Sabitova et al. 2023), Khanty-Mansiysk (Popov 1967), Surgut (Petukhov et al. 2018), Novosibirsk Oblast (Novosibirsk and Toguchinsky District) (Mal’kova and Bogdanov 2004), Tyumen Oblast – Nyzhnevartovsk (Starikov et al. 2017a), Kurgan Oblast (Starikov and Starikova 2021), Salekhard (Starikov et al. 2017a), Tomsk Oblast (Chainsky District) (Mal’kova and Bogdanov 2004), Kemerovo Oblast, Altai Krai, Altai Republic (Bogdanov and Yakimenko 2016), Krasnoyarsk Krai – Podkamennaya Tunguska River and the rural locality Bolshoy Kemchug (Voltsyt 1997). **Ukraine**: Volyn Polesie (Adamovich 1968), outskirts of Kyiv (Akimov and Nebogatkin 2013), Cherkassy Oblast (Nikitchenko 2011), the North-Western seacoast of the Black Sea (Rusev 2009). **Belarus**: throughout the whole territory (Subbotina and Osmolovsky 2022). **Moldova**: reedbeds of the lower reaches of the Prut River (Uspenskaya et al. 1984). **Kazakhstan**: Jambyl Region (Galuzo 1950), Jetisu Region – outskirts of Taldykorgan and Jarkent, Almaty Region – outskirts of Sarkand and Almaty (Golov 1933; Sorokoumov 1937; Ushakova and Fedosenko 1972; Ushakova et al. 1976). **Kyrgyastan**: outskirts of Bishkek, Tokmak Reserve (Grebenyuk 1966), Chuy Valley (Kharadov et al. 2013).

###### Ecology and other information.

*Ixodesapronophorus* has a wide distribution in the Northern Palearctic from the Atlantic coast to Eastern Siberia. Its geographical range generally coincides with the distribution of the water vole, its most frequent host, as both the tick and its common host prefer swampy and humid places for living, especially near water bodies.

##### ﻿ Ixodes eldaricus

Taxon classificationAnimaliaIxodidaIxodidae

Djaparidze, 1950

FBD4A4C3-0D69-504B-8BC2-40FA67CA84B8

Ixodes eldaricus Dzhaparidze, 1950: 117.
Ixodes tatei Arthur, 1959: 108; Clifford et al. 1973: 489.

###### Recorded hosts.

**Aves**: *Alectorischukar* (Gray) (chukar partridge), *Anthuscampestris* (Linnaeus) (tawny pipit), *Athenenoctua* (Scopoli) (little owl), *Chroicocephalusridibundus* (Linnaeus) (black-headed gull), *Coccothraustescoccothraustes* (Linnaeus) (hawfinch), *Coloeusmonedula* (Linnaeus) (western jackdaw), *Currucacommunis* (Latham) (common whitethroat), *Emberizabruniceps* Brandt (red-headed bunting), *Galeridacristata* (Linnaeus) (crested lark), *Lullulaarborea* (Linnaeus) (woodlark), *Lusciniasvecica* (Linnaeus) (bluethroat), *Melanocoryphabimaculata* (Ménétrés) (bimaculated lark), *Monticolasolitarius* (Linnaeus) (blue rock thrush), *Oenanthe* sp. (wheatear), *Passerdomesticus* (Linnaeus) (house sparrow), *Perdixperdix* (grey partridge), *Petroniapetronia* (Linnaeus) (rock sparrow), *Phoenicuruserythronotus* (Eversmann) (Eversmann’s redstart), *Phylloscopusgriseolus* (Blyth) (sulphur-bellied warbler), *Picapica* (Linnaeus) (Eurasian magpie), *Sittatephronota* Sharpe (Eastern rock nuthatch), *Turdusmerula* (common blackbird) (Filippova 1977).

**Mammalia**: *Crociduraleucodon* (bicolored shrew), *Merionespersicus* (Blanford) (Persian jird), *Musmusculus* (house mouse), *Nesokiaindica* (Gray) (short-tailed bandicoot rat), grey dwarf hamster *Nothocricetulusmigratorius* (Pallas), *Rattuspyctoris* (Hodgson) (Turkestan rat), *Rhinolophusmehelyi* (Mehely’s horseshoe bat) (Filippova 1977).

###### Recorded locations

**Figure 7.**
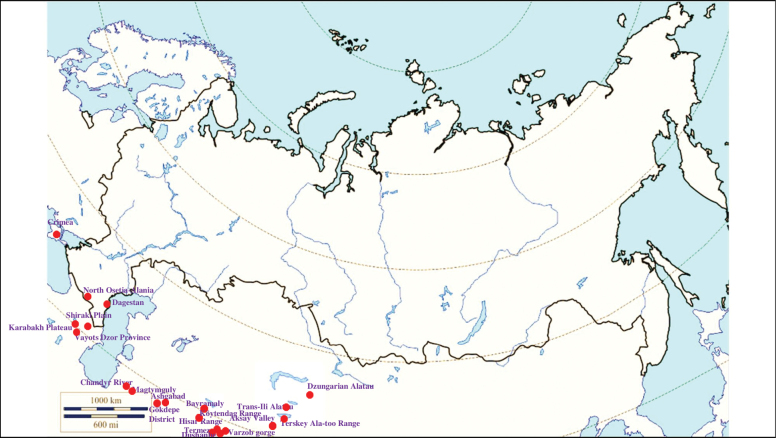
Map of Russia and neighboring countries showing the locations where *Ixodeseldaricus* was reported.

**(Fig. 7). Russia**: Dagestan and North Osetia-Alania (Shatas 1957; Filippova 1977). **Ukraine**: Crimean Peninsula, in particular the Tarkhankut Peninsula and the Kara Dag (Filippova 1974). **Georgia**: the Shiraki Plain and the Vashlovani Nature Reserve (Djaparidze 1950, 1960). **Armenia**: Vayots Dzor Province – the rural locality Herher (Ogandzhanyan 1959). **Azerbaijan**: Karabakh Plateau – Lachin District and Hadrut District, Adzhynokhur Steppe (Ogandzhanyan 1959; Filippova 1977). **Kazakhstan**: Dzungarian Alatau (Ushakova et al. 1976) and Trans-Ili Alatau (Filippova 1977). **Kyrgyzstan**: Terskey Ala-too Range (Filippova 1974). **Turkmenistan**: the Kopet Dagh – the valley of the Chandyr River, Magtymguly, Gökdepe District, outskirts of Ashgabad, Köytendag Range, Bayramaly (Kerbabaev 1960; Kochkareva et al. 1971; Berdyev 1973; Scherbinina 1973). **Uzbekistan**: Termez (Filippova 1977). **Tajikistan**: Hisar Range, Varzob gorge, outskirts of Dushanbe (Filippova 1977).

###### Ecology and other information.

*Ixodeseldaricus* is a little studied endophilic tick species which is mainly a parasite of ground-feeding birds although nymphs and larvae, besides birds, were also found on small mammals – rodents and shrews. It usually inhabits deciduous mountain forests and shrub thickets in mountain river valleys. The vertical distribution range of its occurrence varies from 300 (Ashgabat) to 1800 m (Terskey Ala-too Range and Hisar Range) a. s. l. (Filippova 1977).

Briefly described by a female from the east of Georgia (type locality: the Shiraki Plain), *I.eldaricus* was later found in Armenia and Azerbaijan, and the male, nymph descriptions were based on the material from Azerbaijan (Ogandzhanyan 1959). The holotype female described from the grey partridge is stored at the Institute of Zoology of Ilia State University. The above findings from post-Soviet territories are known from the Crimea, as well as the Causasus and Central Asia. The majority of samples are stored at the collection of the Zoological Institute of the Russian Academy of Sciences.

Additionally, it is important to note that in Crimea this tick species is considered disappearing (Nebogatkin 1998) due to anthropogenic pressure followed by destruction of its habitats and decline in its host populations (Uspensky 2021).

##### ﻿ Ixodes kashmiricus

Taxon classificationAnimaliaIxodidaIxodidae

Pomerantsev, 1948

87E30AFF-1BDC-5A21-9385-E47D351A1C39

Ixodes kaschmiricus Pomerantsev, 1948: 132; Filippova 1969: 675.
Ixodes persulcatus kaschmiricus Pomerantsev, 1948: 132; Filippova 1969: 675.

###### Recorded hosts.

**Mammalia**: *Apodemussylvaticus* (wood mouse), *Canisfamiliaris* Linnaeus (dog), *Ovisaries* Linnaeus (sheep) (Filippova 1977).

###### Recorded locations

**Figure 8.**
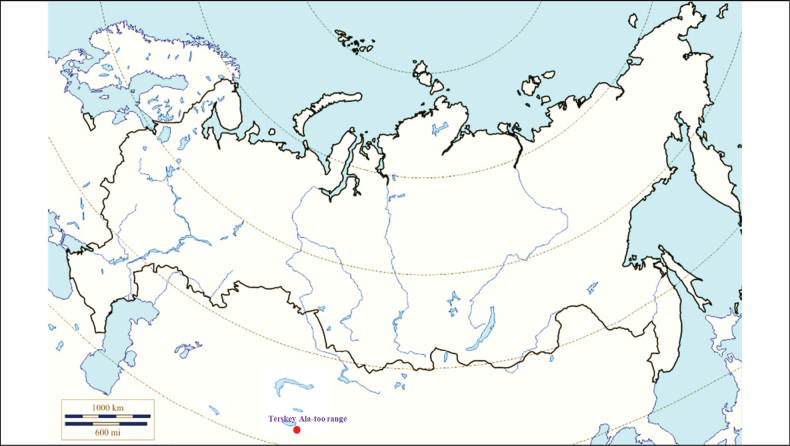
Map of Russia and neighboring countries showing the locations where *Ixodeskashmiricus* was reported.

**(Fig. 8). Kyrgyzstan**: the Tien Shan – northern and eastern slopes of the Terskey Ala-too range (gorges Ulken-Kokpak and Chon-Dzhargylchak) (Filippova 1969).

###### Ecology and other information.

*Ixodeskashmiricus* is a tick species with a disjunctive relict range limited by the Tien Shan in Kyrgyzstan as well as India (Filippova 1977) and Pakistan (Numan et al. 2022). In Kyrgyzstan the tick was found mainly in the mid-altitude vertical zone of the mountains at the lower border of the forest at the altitude of 2000 and 2500 m a. s. l. Cases of parasitism on humans have been recorded (Hoogstraal 1970).

Phylogenetic analysis of mitochondrial and nuclear genes showed that *I.kashmiricus* belongs to the *I.ricinus* group (Kovalev et al. 2018) and clusters with such members of the *I.ricinus* group as *I.apronophorus* and *I.kazakstani* (Numan et al. 2022).

The type specimens are stored at the Zoological Institute of the Russian Academy of Sciences and include the lectotype - female; [India], Kashmir, Vardvan Maru River, northern tributary of Chinab River, 10–13. V.1910, coll. S.P. Trubetskoi; AL I533, as well as the paralectotype - male; AL 533a. *Ixodeskashmiricus* (see: Filippova 1969: 677). Description – Filippova 1977: 292–296 (female, male, nymph, larva) (Filippova 2008). Originally the tick was named *I.persulcatuskaschmiricus* (lapsus).

##### ﻿ Ixodes kazakstani

Taxon classificationAnimaliaIxodidaIxodidae

Olenev & Sorokoumov, 1934

4C85AE4E-D51C-56C0-8737-A75E38704C59

###### Recoeded hosts.

**Mammalia**: *Apodemussylvaticus* (wood mouse) (Filippova 1977), *Canisfamiliaris* (domestic dog) (Kovalev et al. 2018), *Dryomysnitedula* (Pallas) (forest dormouse), *Lepustolai* Pallas (tolai hare), *Musmusculus* (house mouse), *Nothocricetulusmigratorius* (grey dwarf hamster) (Filippova 1977).

**Aves**: *Phasianuscolchicus* Linnaeus (common pheasant) (Filippova 1977).

###### Recorded locations

**Figure 9.**
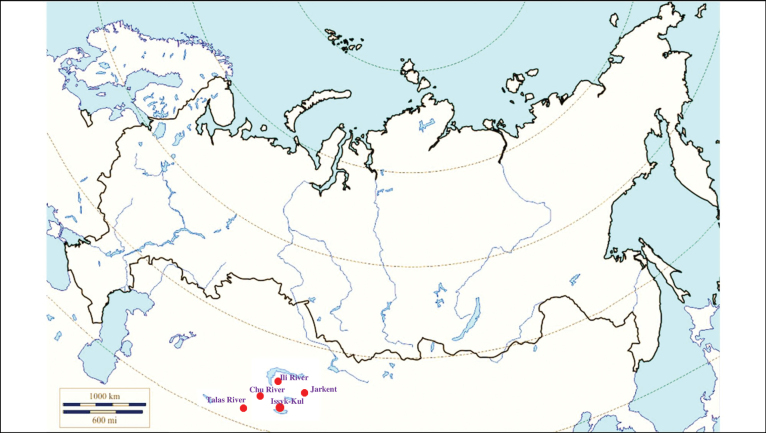
Map of Russia and neighboring countries showing the locations where *Ixodeskazakstani* was reported.

**(Fig. 9). Kazakhstan**: Betpak-Dala – the valley of the Chu River (Ushakova 1961), Tian Shan – the valley of the Ili River (Ushakova 1958; Kovalev et al. 2018), outskirts of Jarkent (Olenev and Sorokoumov 1934; Pomerantsev 1950). **Kyrgyzstan**: the Issyk-Kul basin (Filippova 1958b; Kovalev et al. 2018), the valley of the Talas River (Olenev and Sorokoumov 1934; Pomerantsev 1950; Grebenyuk 1966; Lyashko 1973; Kovalev et al. 2018).

###### Ecology and other information.

*Ixodeskazakstani* is a tick species with a disjunctive relict range limited by Southeastern Kazakhstan and neighboring territories of Kyrgyzstan (Filippova 1977). The patchy arrangement of its range can be explained, above all, by associations of this tick mainly with the animals dwelling in tugai forests which also create humidity conditions in the soil suitable for this tick species (Filippova 1958b). Also, there are some cases of parasitism on livestock and humans (Lyashko 1973; Filippova 1977). On livestock it was found in few numbers among mass parasitism of other tick species.

Phylogenetic analysis of mitochondrial and nuclear genes showed that *I.kazakstani* belongs to the *I.ricinus* group (Kovalev et al. 2018) and clusters with such members of the *I.ricinus* group as *I.apronophorus* and *I.kashmiricus* (Numan et al. 2022). *Ixodeskazakstani* can presumably exemplify links between Nearctic and Palearctic species, so further studies of genetic sequences of *I.kazakstani* are necessary to understand better evolutionary connections between more tick species in the *I.ricinus* group.

The type specimens are stored at the Zoological Institute of the Russian Academy of Sciences and include the holotype: female; Kazakhstan, Jarkent, collected from human dress, 20.VI.1932, coll. Kirin; AL I536. Description - Filippova 1977: 283–290 (female, male, nymph, larva) (Filippova 2008).

##### ﻿ Ixodes laguri

Taxon classificationAnimaliaIxodidaIxodidae

Olenev, 1929

5916BE8F-9804-5A3B-9B6A-E524612C7367

Ixodes laguri Olenev, 1929a: 489.
Ixodes redikorzevi lagurae Olenev: Olenev 1931b: 62.
Ixodes laguri armeniacus Kirshenblat, 1938: 46; Morel and Pérez 1978: 201.
Ixodes laguri colchicus Pomerantsev, 1946: 1; Morel and Pérez 1978: 201.
Ixodes laguri slovacicus Cerny, 1960: 178; Morel and Pérez 1978: 201.

###### Recorded hosts.

**Mammalia**: *Allactagamajor* (Kerr) (great jerboa), *Allocricetuluseversmanni* (Brandt) (Eversmann’s hamster), *Apodemussylvaticus* (wood mouse), *Chionomysnivalis* (European snow vole), *Cricetuscricetus* (European hamster), *Dryomysnitedula* (forest dormouse), *Ellobiustalpinus* (Pallas) (northern mole vole), *Erinaceuseuropaeus* (European hedgehog), *Glisglis* (Linnaeus) (European edible dormouse), *Hemiechinusauratus* (Gmelin) (long-eared hedgehog), *Laguruslagurus* (Pallas) (steppe lemming), *Marmotabobak* (Müller) (bobak marmot), *Martesmartes* (Linnaeus) (European pine marten), *Microtusarvalis* (common vole), *Microtussocialis* (social vole), *Melesmeles* (Linnaeus) (European badger), *Merionesmeridianus* (Pallas) (midday jird), *Mesocricetusbrandti* (Pallas) (Turkish hamster), *Mesocricetusraddei* (Nehring) (Ciscaucasian hamster), *Musmusculus* (house mouse), *Mustelaeversmanii* (Lesson) (steppe polecat), *Mustelanivalis* (least weasel), *Nothocricetulusmigratorius* (grey dwarf hamster), *Pygeretmuspumilio* (Kerr) (dwarf fat-tailed jerboa), *Rattusrattus* (black rat), *Spalaxmicrophthalmos* Gueldenstaedt (greater blind mole-rat), *Spermophiluscitellus* (Linnaeus) (European ground squirrel), *Spermophilusfulvus* (Lichtenstein) (yellow ground squirrel), *Spermophiluspygmaeus* (Pallas) (little ground squirrel), *Spermophilussuslicus* (speckled ground squirrel), *Spermophilusxanthoprymnus* (Bennett) (Asia Minor ground squirrel), *Stylodipustelum* (Lichtenstein) (thick-tailed three-toed jerboa), *Vormelaperegusna* (Güldenstädt) (marbled polecat), *Vulpescorsac* (Linnaeus) (Corsac fox), *Vulpesvulpes* (red fox) (Filippova 1977).

###### Recorded locations

**Figure 10.**
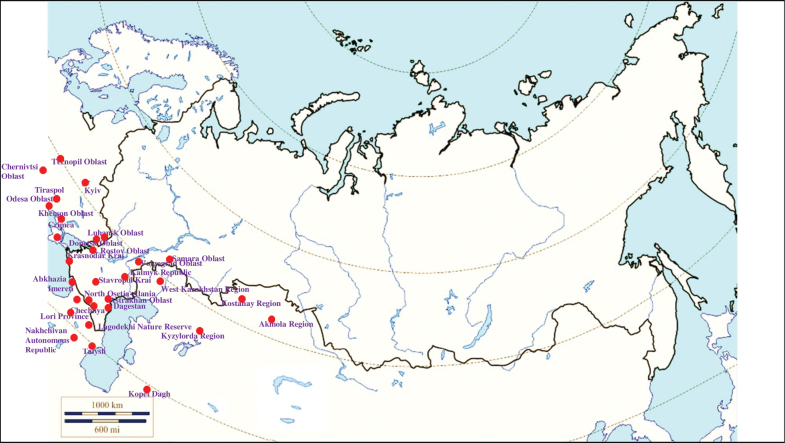
Map of Russia and neighboring countries showing the locations where *Ixodeslaguri* was reported.

**(Fig. 10). Russia**: Samara Oblast (Kirillova and Kirillov 2008b), Rostov Oblast (Stakheev and Panasyuk 2016), Krasnodar Krai (Popov et al. 2019), Stavropol Krai (Tsapko 2019), Volgograd Oblast, Astrakhan Oblast (Nelzina et al. 1955), Kalmyk Republic (Sandzhiev et al. 2006), Chechnya (Baisarova 2021), Dagestan (Musaev et al. 2019) and North Osetia-Alania (Filippova 1977). **Ukraine**: Kyiv (Omeri and Moysak 2013), Odesa Oblast (Rusev 2008), Kherson Oblast, Chernivtsi Oblast, Ternopil Oblast, Luhansk Oblast, Donetsk Oblast, the Crimean Peninsula, particularly in the Syvash (Filippova 1958a; Emchuk 1960; Sklyar 1970; Andryushchenko et al. 2005; Evstafiev 2017). **Moldova**: Bălți Steppe, Bugeac Steppe (Filippova 1977) and Tiraspol (Kravchenko 2014). **Georgia**: Abkhazia (Shaposhnikova and Sakhno 2012), Imereti (Sukhiashvili et al. 2020), Lagodekhi Nature Reserve (Djaparidze 1960). **Armenia**: Lori Province –Nalband and the valley of the river Hrazdan (Filippova 1977). **Azerbaijan**: Talysh (Pomerantsev 1950), the Nakhchivan Autonomous Republic – the Zangezur Mountains (Kadatskaya and Shirova 1963; Filippova 1977). **Kazakhstan**: West Kazakhstan Region (Pomerantsev 1950; Levit 1957; Filippova 1977), Kyzylorda Region (Loseva 1963), Kostanay Region, Akmola Region (Ushakova 1961, 1962). **Turkmenistan**: the Kopet Dagh (Kerbabaev 1961).

###### Ecology and other information.

*Ixodeslaguri* is a tick species which is mainly a nidicolous parasite of rodents and small and medium carnivores, first of all ground squirrels. It is present usually in zonal and mountainous steppes at the altitude of 1500 m a.s.l. This tick species is less common in desert and semi-desert biotopes (Filippova 1977).

Filippova (1977) states that the tick has four subspecies – *I.lagurilaguri*, *I.l.armeniacus*, *I.l.colchicus* and *I.l.slovacicus*. The differential characters of the female and the male of *I.l.slovacicus* are based on comparison with characters of the other subspecies in Pomerantsev (1950) but some of them, such as the genital aperture and chaetotaxy of the scutum and the hypostome and the coxa 1, are not characterized precisely enough (Filippova 1977).

According to Filippova (1977), *I.lagurilaguri* can be found in Moldova, Ukraine, Kazakhstan, as well as in the south of Russia; *I.l.armeniacus* is distributed in the Caucasus – North Osetia-Alania, Dagestan, Georgia, Armenia and Azerbaijan; *I.l.colchicus* is known from the western spurs of the Greater Caucasus, the now abandoned rural locality Babuk-Aul; *I.l.slovacicus* was described from the south-east of Slovakia.

The type specimens of *I.laguri* are deposited at the Zoological Institute of the Russian Academy of Sciences and include *I.l.armeniacus*: the lectotype, female; Armenia, Nalband, from *Mesocricetusbrandti* Nehr., 9.9.1936; AL I558 and the paralectotype, male; AL I556, description – Filippova 1977: 384 (female, male, nymph; larva unknown). (Filippova 2008), as well as *I.l.colchicus*: the lectotype, male; Western Caucasus, near Babuk-Aul, *Glisglis* L., 30.9.1935, coll. V. K. Popov, det. В. Pomerantsev: *I.l.colchicus*, type; AL I554a; paralectotypes: 2 females; AL I554a, description – Filippova 1977: 384 (female, male; nymph and larva unknown) (Filippova 2008).

##### ﻿ Ixodes nipponensis

Taxon classificationAnimaliaIxodidaIxodidae

Kitaoka & Saito, 1967

2DF58F29-958D-587F-9CCE-648A6290F7DF

###### Recorded hosts.

**Mammalia**: *Apodemusagrarius* (striped field mouse), *Craseomysrufocanus* (grey red-backed vole), *Microtusfortis* (Büchner) (reed vole), *Myodesrutilus* (northern red-backed vole) (Filippova 1977).

###### Recorded locations

**Figure 11.**
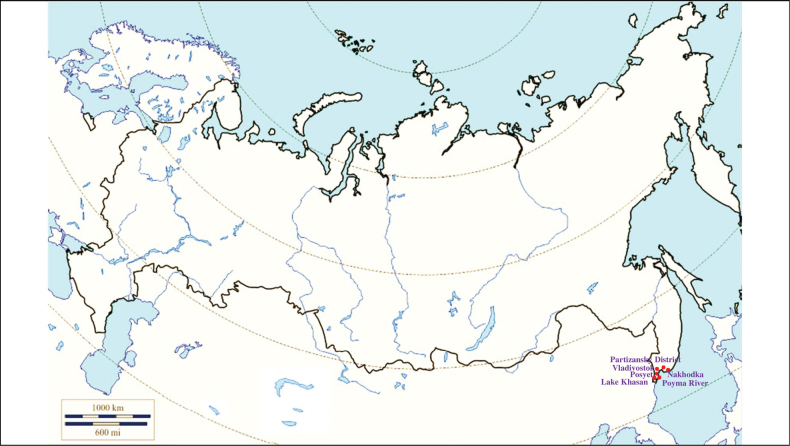
Map of Russia and neighboring countries showing the locations where *Ixodesnipponensis* was reported.

**(Fig. 11). Russia**: Primorsky Krai – the Lake Khasan, the Poyma River, the Partizansky District, outskirts of urban localities Posyet, Kraskino, Slavyanka and cities Vladivostok and Nakhodka, near the village Rechitsa (Filippova 1969; Filippova and Belyaev 1970; Allenov et al. 2015).

###### Ecology and other information.

*Ixodesnipponensis* is a tick species found in Russia in the south and south-west of the Primorsky Krai and also in the Korean peninsula and Japan (Filippova 1977). In Russia it was reported mainly from murine rodents, although in the Republic of Korea it was also observed on lizards (Kim et al. 2018) and cattle, goats, dogs, horses, and birds in Japan (Kitaoka and Saito 1967; Yamaguti et al. 1971).

Multiple cases of parasitism on humans have been recorded (Nakatsukase and Hatsushika 1985; Paik et al. 1989; Cho et al. 1995; Chu et al. 1997; Ryu et al. 1998; Ko et al. 2002).

##### ﻿ Ixodes occultus

Taxon classificationAnimaliaIxodidaIxodidae

Pomerantsev, 1946

20506EED-99D6-5DCF-8395-1EAC47B77CF0

###### Recorded hosts.

**Mammalia**: *Crocidurasuaveolens* (lesser white-toothed shrew), *Diplomesodonpulchellum* (Lichtenstein) (piebald shrew), *Merioneslibycus* Lichtenstein (Libyan jird), *Merionesmeridianus* (midday jird), *Merionespersicus* (Persian jird), *Mustelanivalis* (least weasel), *Nothocricetulusmigratorius* (grey dwarf hamster), *Rhombomysopimus* (Lichtenstein) (great gerbil), *Spermophilopsisleptodactylus* (Lichtenstein) (long-clawed ground squirrel), *Vormelaperegusna* (marbled polecat) (Filippova 1977).

**Reptilia**: *Gloydiushalys* (Pallas) (Halys pit viper) (Filippova 1977).

###### Recorded locations

**Figure 12.**
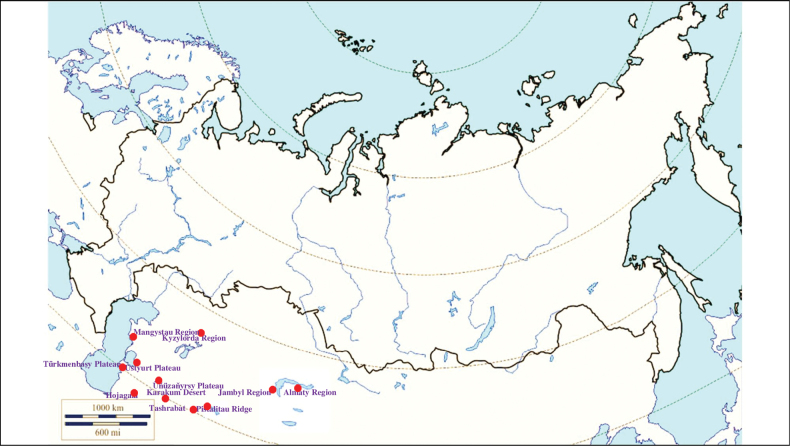
Map of Russia and neighboring countries showing the locations where *Ixodesoccultus* was reported.

**(Fig. 12). Kazakhstan**: Mangystau Region – the Mangyshlak Peninsula (Kaluzhenkova et al. 1961) and the Ustyurt Plateau; Kyzylorda Region (Filippova 1958a; Loseva 1963; Maslennikova and Ushakova 1971), Jambyl Region – the Moiynkum Desert (Maslennikova and Ushakova 1971), Almaty Region – the foothills of the Dzungarian Alatau: the Sholak and Katutau mountains, the deserts Taukum and Saryesik-Atyrau (Ushakova 1960; Maslennikova et al. 1964; Ushakova et al. 1976). **Turkmenistan**: distributed everywhere – the southern Ustyurt, the Octumkumy Desert, the Üňüzaňyrsy and Türkmenbaşy Plateau, the Meshed and Saynaksan Desert, the Karakum Desert (Pomerantsev 1950; Kerbabaev 1961; Kochkareva et al. 1971); Hojagala (Berdyev and Annaev 1997). **Uzbekistan**: the Pistalitau Ridge and the rural locality Tashrabat (Maslennikova and Ushakova 1971).

###### Ecology and other information.

*Ixodesoccultus* is a tick species inhabiting deserts. It is mainly a nidicolous parasite of gerbils and jirds (subfamily Gerbillinae), first of all, the great gerbil, as well as of those small mammals which also use long and deep burrows of great gerbils as shelters (Filippova 1977). Some predators which have strong trophic relationships with gerbils and regularly contact with their colonies act as secondary hosts for this tick species.

The type specimen of *I.occultus* is deposited at the Zoological Institute of the Russian Academy of Sciences and includes the holotype: male; Turkmenia, Repetek, *Rhombomysopimus*, 5.10.1937, coll. B.I. Pomerantsev, type; AL I550. Description – Filippova 1977: 365–371 (female, male, nymph, larva) (Filippova 2008).

##### ﻿ Ixodes pavlovskyi

Taxon classificationAnimaliaIxodidaIxodidae

Pomerantsev, 1946

6474E80F-C719-57D9-A0EB-F4E4B63BCB9D

###### Recorded hosts.

**Aves**: *Acrocephalusdumetorum* Blyth (Blyth’s reed warbler), *Acrocephalusschoenobaenus* (Linnaeus) (sedge warbler), *Anasplatyrhynchos* Linnaeus (mallard), *Anthustrivialis* (tree pipit), *Calliopecalliope* (Pallas) (Siberian rubythroat), *Cardueliscarduelis* (European goldfinch), *Carpodacuserythrinus* (Pallas) (common rosefinch), *Chlorischloris* (Linnaeus) (European greenfinch) *Columbalivia* Gmelin (rock dove), *Corvuscornix* Linnaeus (hooded crow), *Corvuscorone* Linnaeus (carrion crow), *Coturnixcoturnix* (Linnaeus) (common quail), *Crexcrex* (Linnaeus) (corn crake), *Currucacommunis* (Latham) (common whitethroat), *Currucacurruca* (Linnaeus) (lesser whitethroat), *Cyanopicacyanus* Pallas (azure-winged magpie), *Emberizacalandra* Linnaeus (corn bunting), *Emberizacitrinella* Linnaeus (yellowhammer), *Emberizaleucocephalos* Gmelin (pine bunting), *Emberizaspodocephala* Pallas (black-faced bunting), *Ficedulahypoleuca* (Pallas) (European pied flycatcher), *Fringillacoelebs* Linnaeus (Eurasian chaffinch), *Fringillamontifringilla* Linnaeus (brambling), *Laniuscollurio* Linnaeus (red-backed shrike), *Locustellalanceolata* (Temminck) (lanceolated warbler), *Luscinialuscinia* (Linnaeus) (thrush nightingale), *Lusciniasvecica* (Linnaeus) (bluethroat) *Parusmajor* Linnaeus (great tit), *Passermontanus* (Linnaeus) (Eurasian tree sparrow), *Pastorroseus* (Linnaeus) (rosy starling), *Phoenicurusphoenicurus* (Linnaeus) (common redstart), *Phylloscopusfuscatus* (Blyth) (dusky warbler), *Phylloscopustrochiloides* (Sundevall) (greenish warbler), *Picapica* (Eurasian magpie), *Sittaeuropaea* Linnaeus (Eurasian nuthatch), *Sturnusvulgaris* Linnaeus (common starling), *Sylviaborin* (garden warbler), *Tetraourogallus* (western capercaillie), *Tetrastesbonasia* (hazel grouse), *Turdusiliacus* Linnaeus (redwing), *Turdusphilomelos* Brehm (song thrush), *Turduspilaris* Linnaeus (fieldfare), *Turdusruficollis* Pallas (red-throated thrush), *Turdusviscivorus* (mistle thrush) (Filippova 1977; Moskvitina et al. 2014).

**Mammalia**: *Alexandromysoeconomus* (tundra vole), *Apodemusagrarius* (striped field mouse), *Arvicolaamphibius* (European water vole), *Craseomysrufocanus* (grey red-backed vole), *Cricetuscricetus* (European hamster), *Eutamiassibiricus* (Siberian chipmunk), *Lepustimidus* (mountain hare), *Microtusagrestis* (short-tailed field vole), *Microtusarvalis* (common vole), *Musmusculus* (house mouse), *Myodesglareolus* (bank vole), *Myodesrutilus* (northern red-backed vole), *Neomysfodiens* (Eurasian water shrew), *Nothocricetulusmigratorius* (grey dwarf hamster), *Ochotonaalpina* (Alpine pika), *Sciurusvulgaris* (red squirrel), *Sicistabetulina* (northern birch mouse), *Sicistasubtilis* (Pallas) (southern birch mouse), *Sorexaraneus* (common shrew), *Sorexminutus* (Eurasian pygmy shrew), *Sorexroboratus* (flat-skulled shrew), *Stenocraniusgregalis* (Pallas) (narrow-headed vole) (Filippova 1977).

###### Recorded locations

**Figure 13.**
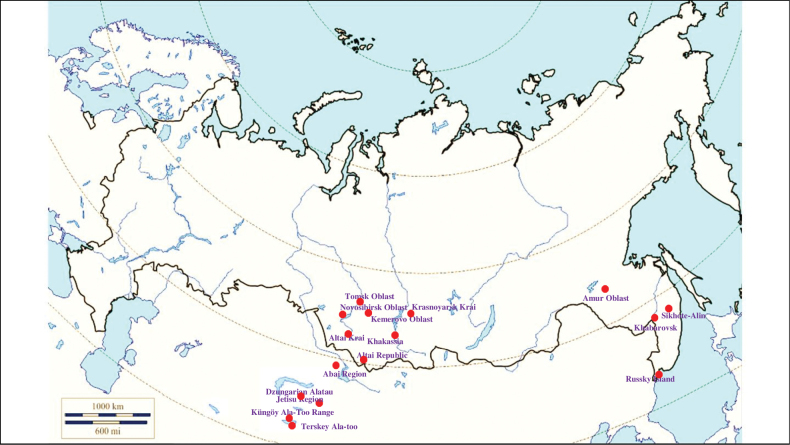
Map of Russia and neighboring countries showing the locations where *Ixodespavlovskyi* was reported.

**(Fig. 13). Russia**: Tomsk Oblast (Kovalev et al. 2015), Novosibirsk Oblast, Altai Republic (Tkachev et al. 2017), Altai Krai, Kemerovo Oblast, Krasnoyarsk Krai, Khakassia, northern spurs of the Western Sayan, Amur Oblast, Khabarovsk, Primorsky Krai – the Sikhote-Alin (Filippova 1969; Sapegina and Ravkin 1969; Filippova and Panova 1998), Russky Island (Nikitin et al. 2021). **Kazakhstan**: East Kazakhstan Region (Tkachev et al. 2017; Perfilyeva et al. 2020), Abai Region, Jetisu Region (Filippova 1977), Tarbagatai Mountains, Dzungarian Alatau, Küngöy Ala-Too Range (Ushakova et al. 1976; Filippova and Panova 1998). **Kyrgyzstan**: Küngöy Ala-Too Range (Filippova and Panova 1998), Terskey Ala-too (Fedorova 2017).

###### Ecology and other information.

*Ixodespavlovskyi* is a tick species distributed in Western Siberia, the Far East, Eastern Kazakhstan, and Kyrgyzstan (Filippova 1977; Fedorova 2017), as well as in China (Guo et el. 2016) and Japan (Nakao et al. 1992; Guglielmone et al. 2023). It more often prefers birds as hosts, as well as small mammals although some cases of human and livestock infestation are also recorded. Its preferred habitats include usually coniferous and deciduous forests, undergrowth, as well as motley grass (Filippova 1977).

Often it can be found in the same biotopes together with *I.persulcatus* with complete coincidence of the seasons of activity of both species at each ontogenetic stage (Filippova 1999) and where their hybridization can also occur (Kovalev et al. 2015; Rar et al. 2019).

In certain areas of Siberia *I.pavlovskyi* outnumbers *I.persulcatus* and also other tick species due to the high abundance of ground-feeding birds, especially in urban landscapes with habitats suitable for ticks like parks and cemeteries. So, for example, in the city of Tomsk in Western Siberia *I.pavlovskyi* dominates everywhere in the city and its outskirts (Romanenko 2011). Probably eventually over time *I.persulcatus* was gradually replaced by *I.pavlovskyi* because it is too difficult for adult *I.persulcatus* to find its preferred hosts, namely mammals (Romanenko and Leonovich 2015).

Filippova and Panova (1998) recognize two subspecies in Russian populations of this tick, namely *I.pavlovskyipavlovskyi* and *I.pavlovskyioccidentalis* which differentiation is based on morphological features between western and eastern specimens.

The type specimens of *I.pavlovskyi* are deposited at the Zoological Institute of the Russian Academy of Sciences and include *I.pavlovskyi*, Pomerantsev (Pomerantsev 1946: 11), the holotype: female; [Russia], DVK [Primorskii Terr.], Imanskii Forestry, hazel, 2.9.1932, type; AL I513. Description – Filippova 1977: 305–312 (female, male, nymph, larva); as well as I.pavlovskyisubsp.occidentalis (Filippova and Panova 1998: 396–411 – female, male, nymph, larva) the holotype: female; Russia, western foothills of Kuznetskii Ala Tau, basin of upper Tom River, environs of Mezhdurechensk, from vegetation, flagging, 24.5.1972, coll. E.D. Chigirik, det. N.A. Filippova; AL I1016 and finally I.pavlovskyisubsp.pavlovskyi (Filippova and Panova 1998: 396–411, female, male, nymph, larva), the holotype (the same as the holotype of the species): see *I.pavlovskyi* (Filippova 2008).

##### ﻿ Ixodes persulcatus

Taxon classificationAnimaliaIxodidaIxodidae

Schulze, 1930

BDAE1241-C08F-5E4C-8EA8-0E08CFD31873

Ixodes persulcatus Schulze, 1930: 294.
Ixodes ricinus miyazakiensis Kishida: Morel and Pérez 1978: 201.
Ixodes persulcatus diversipalpis Schulze, 1930: 294; Pomerantsev 1950: 43.
Ixodes persulcatus cornuatus Olenev: Pomerantsev 1950: 43.
Ixodes sachalinensis Filippova: Kolonin 1981: 49.

###### Recorded hosts.

The spectrum of hosts of *I.persulcatus* is extremely broad both systematically and ecologically and includes more than 200 species of mammals and 100 species of birds (Shilova and Clabovskii 1968). Rarely it can parasitize reptiles – lizards of the family Lacertidae (Ravkin 1969). Literally almost all mammals and birds inhabiting various types of forests and their derivative biotopes can act as hosts for *I.persulcatus*. Larvae and nymphs parasitize more often small and medium-sized mammals, such as shrews, hedgehogs, rodents, and lagomorphs, as well as ground-feeding and ground-nesting birds. Adults usually feed on large and medium-sized mammals – ungulates, carnivores, lagomorphs. Humans and domestic animals can also be hosts for this tick species (Filippova 1977).

###### Distribution in Russia and other post-Soviet countries

**Figure 14.**
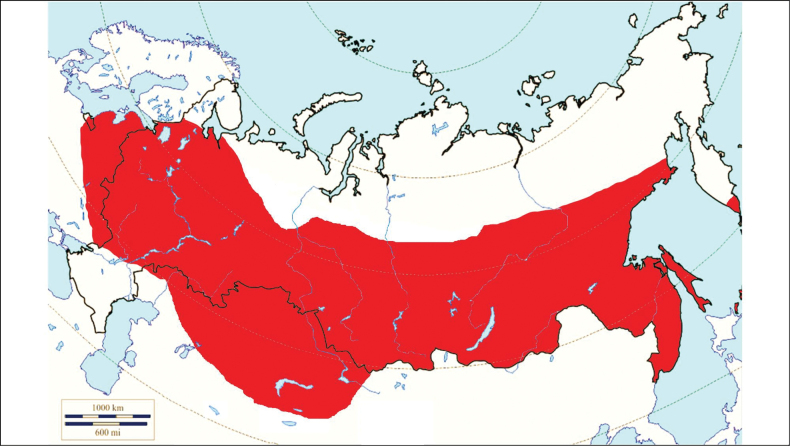
Map of Russia and neighboring countries showing the locations where *Ixodespersulcatus* was reported.

**(Fig. 14).** The range of *I.persulcatus*, like no other Palearctic species, is extended in the latitudinal direction by a continuous strip, covering a significant part of the taiga forest zone in Eurasia between 21°–66° latitude in the northern hemisphere from the Scandinavian Peninsula, the Baltic states, Belarus and Ukraine in the west where it is present sporadically to the east up to the Pacific coast including the Kamchatka Peninsula and the Sakhalin Island and further to the north-east of China, the Korean Peninsula and Japan (Filippova 1977; Wang et al. 2023). This tick belongs to the tick fauna of the next post-Soviet countries: Estonia, Latvia, Lithuania, Belarus, Russia, Ukraine, Kazakhstan, Kyrgyzstan (Guglielmone et al. 2023). The presence of *I.persulcatus* in Ukraine outside the south-west border of the taiga was mentioned by Filippova (1977), although the possibility of permanent populations existing there was disputed by Nebogatkin (1993). Therefore, this probably exemplifies transportation by migratory birds.

###### Ecology and other information.

*Ixodespersulcatus* is an exophilic tick species widely distributed in the northern Palearctic along the forest zone. It may use almost all mammals and birds living in its biotopes; therefore, it is one of the most important vectors of a broad range of tick-borne pathogens. Since it can also transmit tick-borne encephalitis virus, together with *I.ricinus* it has the greatest medical and veterinary significance among other ticks of the genus *Ixodes* in the Palearctic. Another important fact is that *I.persulcatus* is a very aggressive species toward humans (Uspensky 1993) and, therefore, this species represents especially high medical-epidemiological risks.

The most significant part of the range of *I.persulcatus* stretches across the territory of Russia where we can observe the full spectrum of biotopes where *I.persulcatus* can be found. There are a lot of published works about its ecology in different regions which depend on the climatic region and biotic-abiotic conditions in it.

This tick prefers various types of forest and forest-steppe biotopes, especially taiga forests and their derivatives, i.e., mixed forests and bushes (both plain and mountainous), up to 2000 m a.s.l., like in the Tian Shan. In other words, it can inhabit any herbaceous forest and forest-steppe biotope with the level of humidity high enough for reproduction and supporting the life cycle, even in urban landscapes (Filippova 1977). In the Dzungarian Alatau there were some observations of occurring in steppe regions bordering forests and parasitizing the unusual host, namely the grey marmot *Marmotabaibacina* Kastschenko (Bibikov et al. 1961). Permanent and stable populations of *I.persulcatus* exist in some areas adjacent to cities within its range and even inside these cities on condition that the the suitable forest environment together with hosts, such as wild animals of different sizes and stray dogs are present. Examples of such cities are Saint Petersburg, Petrozavodsk, Novosibirsk, Tomsk, Irkutsk, and Vladivostok (Uspensky 2017).

Several studies attest the changing boundaries of the ranges of *I.persulcatus*. It is assumed that ticks of the *I.presulcatus* group appeared and evolved in forest biotopes similar to modern relict forests of the Ussuri type and the taiga of the mountains of Southern Primorye, Southern Siberia, and the Korean Peninsula in the Pliocene. The wide ecological niche of *I.persulcatus* was formed during the formation of the species in the process of its adaptation to various landscape and climatic conditions. This allowed the species to gradually expand its range in the northwestern direction in the Holocene (Filippova 2017). An increase in air temperature by one or several degrees in a particular region near the boundaries of its range was probably the main driver of its expanding distribution. The fact of finding *I.presulcatus* populations in Sweden (Jaenson et al. 2016) and even in the Magadan Oblast in the north-east of Russia where it was absent before (Yamborko et al. 2015) are good examples of the distribution expansion in several directions and confirm the tendency which continues.

In Russia, high numbers of observations show noticeable changes in the distribution of *I.persulcatus* in certain regions. In Karelia the range expansion of *I.persulcatus* to the north is noted in relation to general climate warming (Bugmyrin et al. 2013). A similar observation was also recorded in the Komi Republic (Glushakova et al. 2011). The range expansion of this tick species in Arkhangelsk Oblast and Western and Central Siberia to the north is confirmed both by the results of their records and by the data on tick bites and morbidity in the human population, not only in places which were free from ticks before (Pogodina 2021). Besides that, there are some data about the range expansion of *I.persulcatus* to the north in the Republic of Sakha (Yakutia). The reasons causing these changes are under evaluation but climate change, anthropogenic pressure in natural landscapes as well as the number of vertebrate animals are among the most influential factors. At the same time, it is also possible that inadvertent dispersal of ticks by timber material transported from tick-infested areas may be in part responsible for this phenomenon (Danchinova et al. 2006). Although other factors are not excluded, it is believed that climate changes have made the greatest contribution to the increase in areas primarily for TBE foci in the northern regions of the country. But despite all this, as a result of the same changes, the southwestern part of the range of *I.persulcatus* in Belarus and the Baltic countries has decreased (Pogodina 2021).

Often it can be found in the same biotopes together with *I.ricinus* in Europe and *I.pavlovskyi* in Siberia with complete or partial coincidence of the seasonal activity of these species at each ontogenetic stage (Ushakova and Filippova 1968; Bolotin et al. 1977; Filippova 1999). In zones of sympatry their hybridization can occur, and their hybrids can also transmit tick-borne encephalitis virus and probably other pathogens (Kovalev et al. 2015; Rar et al. 2019; Belova et al. 2023). Under laboratory conditions, interspecific hybridization between *I.ricinus* and *I.persulcatus* was successfully conducted as well. F1 hybrid ticks were completely sterile, as revealed by unsuccessful attempts of their subsequent hybridization with ticks of the parent generation (Balashov et al. 1998). In *I.persulcatus* and *I.ricinus*, any morphological barrier to crossing is undoubdetly absent and then sterility of the F1 hybrid generation is probably a quite significant factor limiting the population size of both species in their sympatric areas. Hybrid ticks also have morphological features allowing to differentiate them at preimaginal and imaginal stages (Bugmyrin et al. 2015, 2016). Moreover, some studies were conducted in the Southern Primorye (Filippova 2002) in sympatric zones of *I.persulcatus* and *I.pavlovskyioccidentalis*, due to the close cohabitation of both species. These showed that in case of these two species there are distinct morphological barriers which are manifested in the fitting of organs involved in mating, in particular their size proportions. According to the result of the studies, mating and hybridization of different tick species are possible only in the next combination: female *I.pavlovskyi* and male *I.persulcatus*. Whereas in case of the reverse combination, the parameters of the genital aperture of the female exceed those of the largest width of the hypostome in the male.

There is an excellent summary on the questing behavior of *I.persulcatus* in the monograph by Filippova (1985). In brief, the ticks climb onto the vegetation in quest of a host. When the host approaches, the tick spreads its first pair of legs and, upon contact with the host, become attached. From time to time, ticks perform vertical migrations and go even into the soil litter for rehydration. Horizontal movements of ticks towards trails used by potential hosts are also possible, as well as crawling onto a nearby animal. Ticks react to humans by spreading their first legs from distances of ~ 15–20 m. At short distances, ticks also react to a heat source. In general, a similar pattern of questing behavior is used by other exophilic ticks of the genus *Ixodes*.

In *I.persulcatus* there is an important signaling mechanism causing a morphogenetic diapause – a developmental delay which is the response of ticks to the duration of the diurnal photoperiod (Belozerov 1976). Moreover, *I.persulcatus* has a behavioral diapause of non-engorged adult ticks, which is not connected with photoperiodic regulation (Korenberg et al. 2021). But as the studies in the Kirov Oblast and Udmurt Republic showed, in more warmer areas, an increased proportion of engorged larvae and nymphs develop without the diapause and the reason for this is the early activation and, as a result, their mass feeding on hosts in the first half of summer. The factors determining the diapause of engorged larvae and nymphs in the compared regions practically do not differ (Korotkov 2008). The correlation of the tick number varies, depending on the type of biotope, as well as temperature and humidity and also many other abiotic factors. For example, in boreal taiga forests of Karelia mainly *I.persulcatus* dominates (except the southwestern part where the mass species is *I.ricinus*) (Bugmyrin et al. 2013). The beginning of adult *I.persulcatus* activity also differs in different regions depending on the sum of abiotic factors listed above. For example, in the Far East the seasonal peak in the number of larvae is observed in the third decade of May – second decade of July, whereas in the European part of its range in the third decade of July (Belozerov 1976; Filippova 1977; Balashov 1998; Korenberg et al. 2013). In the territory from the Volga River to Primorye the average activity of adult ticks varies from 60 to 140 days (Korenberg et al. 1974). The boundaries of the range of the tick are determined mainly by the combination of photo- and hygrothermal factors. The general indicators of warmth and moisture along the range of this tick species vary widely. The fundamental ecological niche of *I.persulcatus* with the broad scope of its preferred conditions allows it to adapt to the wide diversity of biotopes in the forest zone.

Some type specimens of *I.persulcatus* are deposited at the Zoological Institute of the Russian Academy of Sciences and include I.persulcatussubsp.diversipalpis (Schulze 1930: 300), lectotype: male; [Russia, Primorskii Terr.], lower Amur River, 8 km of Vyatskoe Vill., 26.VI.1910, coll. Soldatov, det. N.О. Olenev: *I.ricinusovatus*; AL I266, as well as the paralectotypes: 1 female, 1 male; AL I266a. *I.persulcatus* (see: Filippova 1969: 677). Description – Filippova 1977: 316–327 (female, male, nymph, larva) (Filippova 2008). But Filippova (1969) also states that re-examination of the type material of the above subspecies demonstrated that the specimens used for describing differences of this subspecies are damaged in some morphologically important parts (not noticed before), and the key morphological characters that were previously thought to distinguish the subspecies are not specific enough and can be found in ticks throughout their entire geographical range.

##### ﻿ Ixodes redikorzevi

Taxon classificationAnimaliaIxodidaIxodidae

Olenev, 1927

04DDCEEE-CCEB-584E-B7C4-DB4865532D04

Ixodes redikorzevi Olenev, 1927: 219.

###### Recorded hosts.

**Mammalia**: *Apodemusagrarius* (striped field mouse), *Apodemusmystacinus* (Danford and Alston) (eastern broad-toothed field mouse), *Apodemusuralensis* (Ural field mouse), *Arvicolaamphibius* (European water vole), *Chionomysnivalis* (European snow vole), *Chionomysroberti* (Thomas) (Robert’s snow vole), *Cricetuscricetus* (European hamster), *Crociduraleucodon* (bicolored shrew), *Crocidurasuaveolens* (lesser white-toothed shrew), *Dryomysnitedula* (forest dormouse), *Erinaceuseuropaeus* (European hedgehog), *Glisglis* (European edible dormouse), *Hemiechinusauratus* (long-eared hedgehog), *Lepuseuropaeus* (European hare), *Marmotabobak* (bobak marmot), *Martesmartes* (European pine marten), *Melesmeles* (European badger), *Merioneslibycus* (Libyan jird), *Merionesmeridianus* (midday jird), *Merionespersicus* (Persian jird), *Merionestamariscinus* (Pallas) (tamarisk jird), *Merionestristrami* Thomas (Tristram’s jird), *Mesocricetusauratus* Waterhouse (golden hamster), *Mesocricetusraddei* (Ciscaucasian hamster), *Microtusarvalis* (common vole), *Microtusmajori* (Major’s pine vole), *Microtussocialis* (social vole), *Musmusculus* (house mouse), *Mustelaeversmanii* (steppe polecat), *Mustelanivalis* (least weasel), *Nesokiaindica* (short-tailed bandicoot rat), *Nothocricetulusmigratorius* (grey dwarf hamster), *Rattusnorvegicus* (brown rat), *Rattuspyctoris* (Turkestan rat), *Rattusrattus* (black rat), *Rhombomysopimus* (great gerbil), *Sciurusanomalus* Gmelin (Caucasian squirrel), *Sciurusvulgaris* (red squirrel), *Sicistabetulina* (northern birch mouse), *Sicistasubtilis* (southern birch mouse) *Spalaxgiganteus* Nehring (giant blind mole-rat), *Spalaxmicrophthalmos* Gueldenstaedt (greater blind mole-rat), *Spermophilopsisleptodactylus* (long-clawed ground squirrel), *Spermophiluspygmaeus* (little ground squirrel), *Sorexaraneus* (common shrew), *Vormelaperegusna* (marbled polecat) *Vulpesvulpes* (red fox) (Filippova 1977).

**Aves**: *Alaudaarvensis* Linnaeus (Eurasian skylark), *Alectorischukar* (chukar partridge), *Anthuscampestris* (tawny pipit), *Anthuspratensis* (Linnaeus) (meadow pipit), Coccothraustescoccothraustes (hawfinch), *Columbalivia* (rock dove), *Emberizacalandra* (corn bunting), *Emberizaschoeniclus* (Linnaeus) (common reed bunting), *Erithacusrubecula* (Linnaeus) (European robin), *Galeridacristata* (crested lark), *Garrulusglandarius* (Linnaeus) (Eurasian jay), *Lullulaarborea* (woodlark), *Melanocoryphacalandra* (Linnaeus) (calandra lark), *Mergusserrator* Linnaeus (red-breasted merganser), *Oenanthehispanica* (Linnaeus) (western black-eared wheatear), *Oenantheisabellina* (Temminck) (Isabelline wheatear), *Oenanthelugens* (Lichtenstein) (mourning wheatear), *Oenantheoenanthe* (Linnaeus) (northern wheatear), *Oenanthepicata* (Blyth) (variable wheatear), *Phylloscopuscollybita* (Vieillot) (common chiffchaff), *Phylloscopusfuscatus* (dusky warbler), *Picapica* (Eurasian magpie), *Pteroclesorientalis* (Linnaeus) (black-bellied sandgrouse), *Saxicolatorquatus* (Linnaeus) (African stonechat), *Sturnusvulgaris* (common starling), *Turdusmerula* (common blackbird), *Turdusphilomelos* (song thrush), *Turdusruficollis* (red-throated thrush) (Filippova 1977).

**Reptilia**: *Darevskiachlorogaster* (Boulenger) (greenbelly lizard) (Orlova et al. 2022), *Lacertaagilis* Linnaeus (sand lizard) (Filippova 1977), *Lacertastrigata* Eichwald (Caucasus emerald lizard) (Orlova et al. 2023), *Pseudopusapodus* (Pallas) (Pallas’s glass lizard) (Filippova 1977).

###### Recorded locations

**Figure 15.**
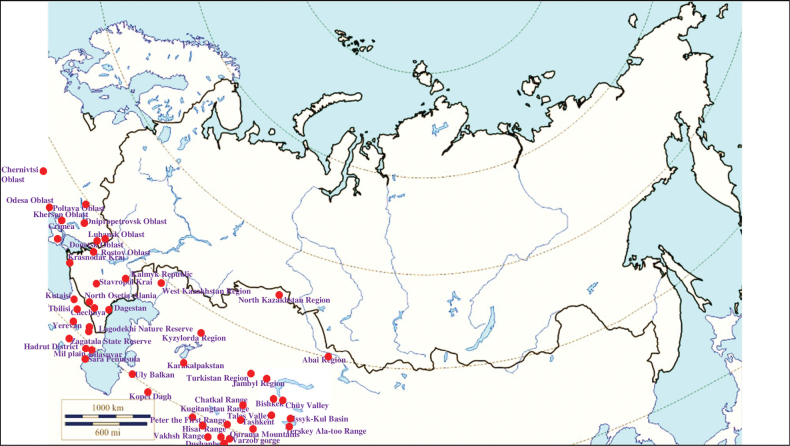
Map of Russia and neighboring countries showing the locations where *Ixodesredikorzevi* was reported.

**(Fig. 15). Russia**: Rostov Oblast (Khametova et al. 2018), Krasnodar Krai, Stavropol Krai, Kalmyk Republic, Chechnya, Dagestan, and North Osetia-Alania (Shatas 1957; Shevchenko et al. 1960; Zaytsev and Popova 1967; Tiflova 1974; Filippova 1977; Abdulmagomedov et al. 2017; Zaytseva et al. 2022). **Ukraine**: Odesa Oblast (Bugeac Steppe), Kherson Oblast (Black Sea Biosphere Reserve), Poltava Oblast, Chernivtsi Oblast, Dnipropetrovsk Oblast, Luhansk Oblast, Donetsk Oblast, widely distributed in the Crimean Peninsula (Emchuk 1960; Emchuk 1967; Sklyar 1970; Filippova 1977). **Moldova**: the north of the country (Uspenskaya et al. 2006). **Georgia**: outskirts of Kutaisi and Tbilisi and the Lagodekhi Nature Reserve, as well as the seacoast of the Black Sea (Kirschenblatt 1936; Djaparidze 1960; Filippova 1977). **Armenia**: outskirts of Yerevan and most of the rest of the territory (Zilfyan et al. 1960; Tiflova 1974). **Azerbaijan**: Zagatala State Reserve, Hadrut District, and the Mil plain (Tiflova 1974), outskirts of the Bilasuvar, the Sara Peninsula (Kirschenblatt 1936), Talysh (Pomerantsev 1950), Nakhchivan Autonomous Republic (Kadatskaya and Shirova 1963; Filippova 1977). **Kazakhstan**: West Kazakhstan Region, Kyzylorda Region, North Kazakhstan Region, Jambyl Region, Turkistan Region, Abai Region (Loseva 1963; Popova and Sokolova 1963). **Kyrgyzstan**: outskirts of Bishkek, Chüy Valley, Talas Valley, Issyk-Kul Basin, Terskey Ala-too Range (Filippova 1958b; Grebenyuk 1966; Filippova 1977). **Turkmenistan**: foothills of the Uly Balkan and the Kopet Dagh; the Kugitangtau Range (Kochkareva et al. 1971; Filippova 1977). **Uzbekistan**: outskirts of Tashkent, foothills of the Chatkal Range, Qurama Mountains, the Hisar Range, the Kugitangtau Range and Karakalpakstan – the Ustyurt Plateau and the lower reaches of the Amu Darya River (Kuklina 1967; Uzakov 1972; Filippova 1977). **Tajikistan**: Hisar Range - Varzob gorge, outskirts of Dushanbe – the Ramit State Nature Reserve, Vakhsh Range, Peter the First Range (Lotozky 1951; Sosnina 1957; Filippova et al. 1966; Kochkareva et al. 1971; Filippova 1977).

###### Ecology and other information.

*Ixodesredikorzevi* is a tick species which is mainly a parasite of rodents, shrews, and small carnivores, as well as of dendrophilic ground-feeding birds and rarely reptiles (Filippova 1977). According to Tiflova (1974), this species is considered exophilic and can be found in significant numbers on dendrophilic birds. In the absence of mammalian and avian hosts, *I.redikorzevi* can parasitize lizards in significant numbers (Orlova et al. 2022). It usually inhabits mountain deciduous forests and steppes located nearby.

Beyond the post-Soviet territories considered above, the range of this tick covers also Eastern Europe, Turkey, Israel, as well as Afghanistan (Filippova 1977) and China (Yin et al. 2010).

At the current moment it is still questionable whether *I.redikorzevi* is a synonym of *I.acuminatus* or not. Kolonin (2009) considers this species a synonym of *I.acuminatus*, but Guglielmone et al. (2010) regard it as provisionally valid. As it was fairly noted by Guglielmone et al. (2014) this question can be solved by comparison of the type specimens of both species. Moreover, Pomerantsev (1950) described by females two subspecies: *I.redikorzeviredikorzevi* and *I.redikorzeviemberizae*. Later the other subspecies *I.redikorzevitheodori* was described although Filippova comments (1977) that the authors had quite little material during descriptions but the differences in size and shape of some characters are visible and it is necessary to compare more specimens from more locations of its large area of distribution.

*Ixodesredikorzeviredikorzevi* occurs in Ukraine, the Transcaucasus and Tajikistan according to Pomerantsev (1950); and *I.redikorzeviemberizae* can be found in Lankaran and the Hisar Range in Tajikistan. Later the other subspecies, *I.redikorzevitheodori* was described from the Middle East (Warburton 1927).

The type specimens of *I.redikorzevi* are deposited at the Zoological Institute of the Russian Academy of Sciences and include the holotype: female; [former] Tavricheskaya Province (Crimea), Yaman-Kala, near Baidar, 25.10.1924, coll. V. Shnitnikov, AL I338 and the paralectotype of *I.redikorzeviemberizae* female; AL I522. Description – Pomerantsev 1950: 63 (female; male unknown); Filippova 1977: nymph, larva (Filippova 2008).

##### ﻿ Ixodes ricinus

Taxon classificationAnimaliaIxodidaIxodidae

(Linnaeus, 1758)

C024D54F-CCC2-5B72-8390-9FF5CE14ED9C

Acarus ricinus Linnaeus, 1758: 616.
Ixodes reduvius (Linnaeus): Neumann 1911: 12.
Ixodes sanguisugus (Linnaeus): Morel and Pérez 1978: 201.
Ixodes vulgaris (Fabricius): Neumann 1911: 12.
Ixodes holsatus (Fabricius): Nuttall and Warburton 1911: 285.
Ixodes megathyreus Leach: Neumann 1911: 12.
Ixodes bipunctatus Risso: Neumann 1911: 12.
Ixodes trabeatus Audouin: Neumann 1911: 12.
Ixodes marginalis Hahn: Oudemans 1896: 191.
Ixodes sciuri Koch: Neumann 1911: 12.
Ixodes fuscus Koch: Neumann 1911: 12.
Ixodes sulcatus Koch: Neumann 1911: 12.
Ixodes rufus Koch: Neumann 1901: 249.
Ixodes lacertae Koch: Neumann 1911: 12.
Ixodes pustularum Mégnin: Neumann 1911: 12.
Ixodes vicinus Yerrill: Oudemans 1896: 191.
Ixodes fodiens Murray: Neumann 1904: 444.
Ixodes nigricans Neumann: Schulze 1939: 1.
Ixodes areolaris Olenev: Pomerantsev 1950: 37.

###### Recorded hosts.

The host spectrum of *I.ricinus* is extremely broad both systematically and ecologically, including literally almost all mammals and birds of its geographical range, rarely even reptiles inhabiting the same biotopes with the tick. The fact of mass parasitism of immature stages on lizards of the Lacertidae family, in particular species of the genus *Darevskia* in the Caucasus (Kidov et al. 2013; Orlova et al. 2022) in habitats where they outnumber small mammals probably brightly demonstrates that *I.ricinus* is a generalist tick capable to use almost any available terrestrial vertebrates as hosts. Overall, the list of hosts consists of more than 300 species of mammals, birds and reptiles which have been recorded (Gern et al. 2002). Humans and domestic animals can also be hosts for the tick (Filippova 1977).

###### Distribution

**Figure 16.**
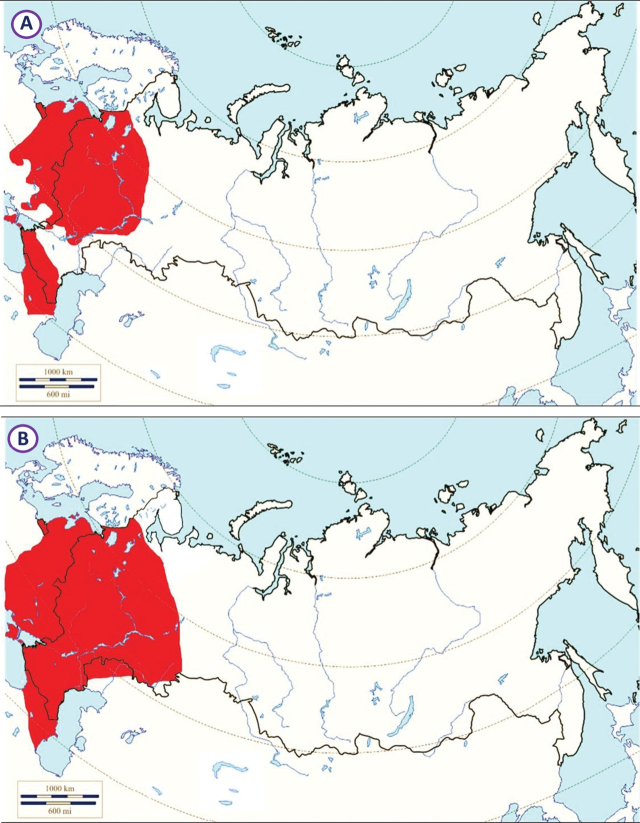
Map of Russia and neighboring countries showing the locations where *Ixodesricinus* was reported: **A** before 1975 **B** from 1976.

**(Fig. 16).** The distribution of *I.ricinus* in Russia includes almost the whole territory of its European part excluding subpolar tundra areas (see the map) (Filippova 1977; Kahl and Gray 2023) and due to climate changes, the distribution of this tick species becomes wider (Gray et al. 2009; Yasyukevich et al. 2009). *Ixodesricinus* is part of the tick fauna of the following post-Soviet countries: Estonia, Latvia, Lithuania, Belarus, Russia, Ukraine, Moldova, Georgia, Azerbaijan, Armenia, Turkmenistan, and Kazakhstan (Guglielmone et al. 2023). In Kazakhstan a little number of specimens were found in the northern part of West Kazakhstan Oblast (Maikanov 2012). In Turkmenistan the tick was also recorded in few numbers in the western foothills of the Kopet-Dag (Kerbabaev 1960) which probably could be transported there by migratory birds.

###### Ecology and other information.

*Ixodesricinus* is an exophilic tick species widely distributed in Europe, mostly inhabiting deciduous and mixed forest zones in both plain and mountainous areas, as well as forest-steppes bordering them. It also occurs in city parks and gardens (Gray 1998). In addition, it can be found in North Africa (Arthur 1965). In Ukraine *I.ricinus* colonized and reached a high abundance in artificial forest plantations of the Askania-Nova Nature Reserve surrounded from all sides by steppes for a period of less than 80 years (Emchuk 1972). In urban areas with conditions able to support tick populations, for example, Minsk or Kyiv, *I.ricinus* usually dominates among other tick species, especially among members of the genus *Ixodes* (Uspensky 2017). This tick species uses almost all forest vertebrate animals as hosts and, together with *I.persulcatus*, it is one of the most important vectors of a broad spectrum of tick-borne pathogens, first of all, tick-borne encephalitis virus (Filippova 1977).

Often it can be found in the same biotope with *I.persulcatus*, often exhibiting complete or partial coincidence of seasonal activity at each ontogenetic stage (Filippova 1999). In zones of sympatry their hybridization can occur, and although hybrid offspring are incapable of reproduction (Bugmyrin et al. 2015), they can still transmit tick-borne encephalitis virus and probably other pathogens (Kovalev et al. 2016; Belova et al. 2023). The absence of any morphological barrier for copulation was discovered in geographical points of probably the secondary sympatric zone (Filippova 2002) of *I.persulcatus* and *I.ricinus* in the north-west of the East European Plain (Balashov et al. 1998). However, in some areas of this sympatric zone, for example, in southern Karelia, its slight shrinking has recently been noted due to the withdrawal of *I.ricinus* from territories where it used to live (Bespyatova and Bugmyrin 2021).

Due to the high epidemiological significance and wide distribution of *I.ricinus* and its regular contacts with humans and domestic animals, its biology and life cycle were more extensively studied than in case of any other species of its genus inhabiting the same territories. As a species, *I.ricinus* probably appeared approximately 8–12 thousand years ago when deciduous and mixed forests formed in the southeast of Europe and the Mediterranean, as well as in the northern and northeastern slopes of the Greater Caucasus, when current environmental conditions of these territories have begun to shape. And the climate there was also milder than in Siberian taiga forests where *I.persulcatus* evolved (Filippova 2017).

It was revealed that in a certain region the duration of tick activity period and the number of adult ticks depend on spring and summer temperatures and air humidity (Korotkov et al. 2015; Korenberg et al. 2021). Females and larvae usually attach to hosts when the air near the soil warms up from +2 to +30 °С, and in the case of nymphs from +2 to +22 °С. The relative humidity of the surrounding air has to be higher than 60% for an extended period of time (Sirotkin and Korenberg 2018). It is absolutely important for ticks to receive the necessary amount of warmth to complete their metamorphosis at each stage within a strictly defined period of time (Korenberg et al. 2013). As a consequence, the seasonal activity of all stages of *I.ricinus* is more extended than in the case of *I.persulcatus*, and engorged ticks begin oviposition or metamorphosis without strict dependance on the photoperiod. Therefore, in the southern range of distribution (the Mediterranean, Central Europe, the Caucasus) ticks initiate activity in the end of March – the beginning of April (Korenberg et al. 2021), whereas in Eastern European regions – in April (Medvedev et al. 2016; Korenberg et al. 2021). *Ixodesricinus* also uses a diapause as a biological mechanism, although due to warmer conditions in the majority of its distribution range, no more than 10–20% of ticks at each stage undergo such an interruption of development (Korenberg and Kovalevsky 1977; Korenberg et al. 2016).

##### ﻿ Ixodes sachalinensis

Taxon classificationAnimaliaIxodidaIxodidae

Filippova, 1971

9A32D181-F543-50D6-A98F-84E74E797DE0

Ixodes sachalinensis Filippova, 1971: 236; Kolonin 1981: 49.
Ixodes persulcatus diversipalpis Schulze, 1930: 294; Pomerantsev 1950: 43.
Ixodes persulcatus cornuatus Olenev: Pomerantsev 1950: 43.

###### Recorded hosts.

**Mammalia**: *Lepustimidus* (mountain hare) (Filippova 1977).

###### Recorded locations

**Figure 17.**
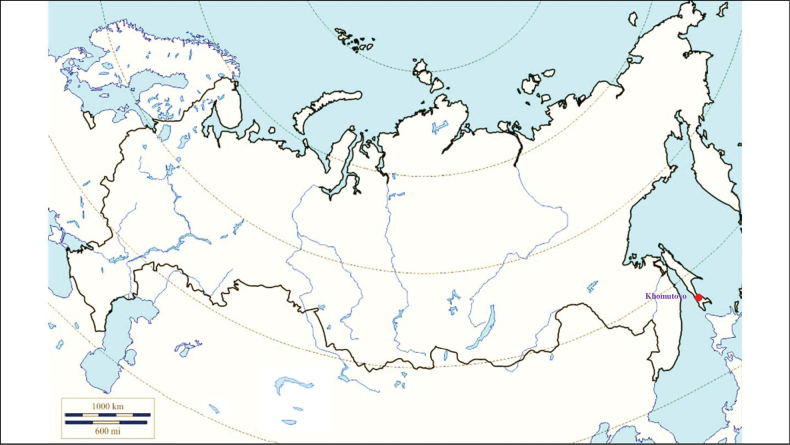
Map of Russia and neighboring countries showing the locations where *Ixodessachalinensis* was reported.

**(Fig. 17). Russia**: the Sakhalin Island, Sachalin Oblast, the rural locality Khomutovo (Filippova 1971).

###### Ecology and other information.

*Ixodessachalinensis* is a tick species known only by the single finding from Sakhalin. It was collected from a mountain hare together with 79 females, 15 males and 7 nymphs of *I.persulcatus* (Filippova 1971).

Kolonin (2009) and Camicas et al. (1998) consider *I.sachalinensis* a synonym of *I.persulcatus*, but Barker and Murrell (2004) and Guglielmone et al. (2009, 2010) recognize this species as valid.

The type specimen is deposited at the Zoological Institute of the Russian Academy of Sciences and includes the holotype: female; [Russia], Sakhalin, near Khomutovo Vill., *Lepustimidus*, 27.5.1950, [coll.: unknown]; AL I729 (Filippova 2008).

#### ﻿Subgenus Ixodiopsis Filippova, 1957: 31.

##### ﻿ Ixodes angustus

Taxon classificationAnimaliaIxodidaIxodidae

Neumann, 1899

5BC69629-C596-5E0D-96AE-86FF27277049

Ixodes angustus Neumann, 1899: 136.

###### Recorded hosts.

**Mammalia**: *Alexandromysoeconomus* (tundra vole), *Craseomysrufocanus* (grey red-backed vole), *Eutamiassibiricus* (Siberian chipmunk), *Musmusculus* (house mouse), *Myodesrutilus* (northern red-backed vole), *Ochotonaalpina* (alpine pika), *Rattusnorvegicus* (brown rat), *Sicistacaudata* Thomas (long-tailed birch mouse), *Sorexaraneus* (common shrew), *Sorexminutus* (Eurasian pygmy shrew) (Filippova 1977).

###### Recorded locations

**Figure 18.**
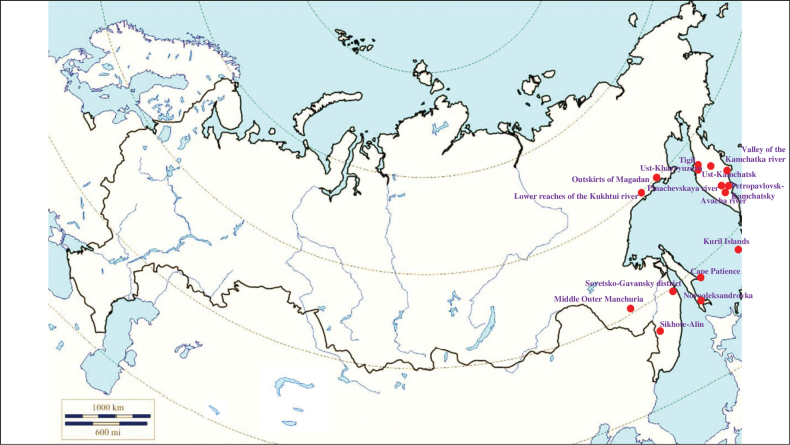
Map of Russia and neighboring countries showing the locations where *Ixodesangustus* was reported.

**(Fig. 18). Russia**: outskirts of Magadan and the lower reaches of the Kukhtui River, Okhotsky district – the northernmost points of record of *I.angustus* in the Palearctic (Belyaev 1963); Kamchatka Peninsula – outskirts of the villages Tigil and Ust-Khayryuzovo (Pomerantsev 1950), the valley of the Kamchatka River to Ust-Kamchatsk (Serdjukova 1956), the eastern coast of the Kamchatka peninsula to Petropavlovsk-Kamchatsky (Speranskaya 1958), the valley of the rivers Avacha and Pinachevskaya (Paramonov et al. 1966); Middle Outer Manchuria (Filippova 1977); Sovetsko-Gavansky district (Emelyanova and Koshkin 1962); Sikhote-Alin (Belyaev and Filippova 1976); Sakhalin – Novoaleksandrovka (former Konuma), the valley of the Lyutoga River (Pomerantsev 1950) and the Cape Patience (Skrynnik 1950; Asanuma 1951; Violovich 1958, 1960; Savitsky and Okuntsova 1967; Timofeeva and Kon’kova 1971); Kuril Islands – Simushir (Pomerantsev 1950; Violovich 1958, 1960; Timofeeva and Kon’kova 1971).

###### Ecology and other information.

*Ixodesangustus* occurs in the Palearctic predominantly on the East Asian coast and also in the Nearctic – Canada and the USA (Filippova 1977). In the Russian Far East in Outer Manchuria, the islands and along the main ridges of the Sikhote-Alin it inhabits a wide range of biotopes: various types of mixed and broad-leaved forests in mountains and valleys, as well as tundra and rocks, stone outcrops, coastal biotopes, meadow and river valleys (Speranskaya 1958; Violovich 1958; Emelyanova and Koshkin 1962; Belyaev 1963; Paramonov et al. 1966; Savitsky and Okuntsova 1967; Belyaev and Filippova 1976).

*Ixodesangustus* is considered a nidicolous ectoparasite of rodents and shrews because it was found not only on hosts but also in their burrows (Filippova 1977), although there are documented cases on this species biting humans without contacts with burrows (Cooley 1946). As a parasite which is connected with rodents, and, like other rodent ticks, *I.angustus* plays a role in supporting natural foci of tick-borne infections such as anaplasmosis (Yamborko and Eremeeva 2014) and the Lyme disease (Peavey et al. 2000).

Although hyperparasitism is not common in *Ixodes* ticks, *I.angustus* belongs to a small number of species of the genus, in which this phenomenon was recorded (Durden et al. 2018), when a male was feeding from a female attached to a red squirrel *Tamiasciurushudsonicus*. The other *Ixodes* species in which males have been recorded to attach and feed on engorging conspecific females include *I.holocyclus* in Australia and *I.pilosus* in South Africa (Oliver et al. 1986).

##### ﻿ Ixodes pomerantzevi

Taxon classificationAnimaliaIxodidaIxodidae

Serdjukova, 1941

8FD3B2BA-3B1C-52FE-A38A-DC650F222086

Ixodes pomerantzevi Serdjukova, 1941: 519.

###### Recorded hosts.

**Mammalia**: *Apodemusagrarius* (striped field mouse), *Craseomysrufocanus* (grey red-backed vole), *Erinaceusamurensis* Schrenk (Amur hedgehog), *Eutamiassibiricus* (Siberian chipmunk), *Microtusfortis* (reed vole), *Myodesrutilus* (northern red-backed vole), *Sorexaraneus* (common shrew) (Filippova 1977), *Sorexcaecutiens* (Laxmann’s shrew), *Sorexunguiculatus* Dobson (long-clawed shrew), (individual specimens ((Okulova et al. 1986), *Rattusnorvegicus* (brown rat), *Tscherskiatriton* (De Winton) (greater long-tailed hamster) (Filippova 1977).

###### Recorded locations

**Figure 19.**
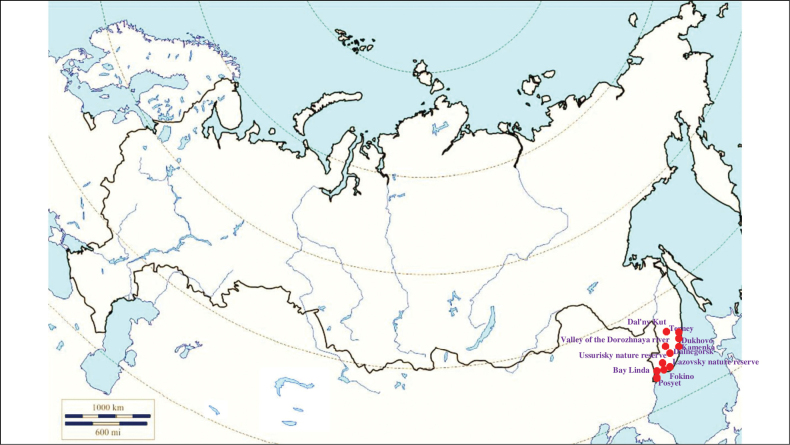
Map of Russia and neighboring countries showing the locations where *Ixodespomerantzevi* was reported.

**(Fig. 19). Russia**: Sikhote-Alin – outskirts of Dal’ny Kut (the northernmost point of finding (Filippova 1977), valley of the Dorozhnaya River, Dalnegorsk, Ussurisky (former Komarovskii) Nature Reserve; coast of the Sea of Japan – outskirts of the villages Terney, Dukhovo, Kamenka, Lazovsky nature reserve, Fokino (former Promyslovka), the bays Razboynik and Linda; the coast of the Peter the Great Gulf: Kedrovaya Pad Nature Reserve, the rural localities Barabash and Posyet (Serdjukova 1941; Pomerantsev 1950; Slonov 1961; Khudyakov 1963; Belyaev and Filippova 1976).

###### Ecology and other information.

*Ixodespomerantzevi* is a relict species occurring on the East Asian coast (Filippova 1977) and in Russia its distribution is limited to a few locations in Outer Manchuria (a.k.a. Primorsky Krai) in the Russian Far East (Tsapko 2020). It is also known to occur in Korea (Kim et al. 2009a, 2010, 2011) and China (Guo et al. 2016). Predominantly it can be found in coniferous and broad-leaf forests, or secondary forests and bush thickets, as well as rock and stone outcrops among trees in the Sikhote-Alin and on the coast of the Sea of Japan (Belyaev and Filippova 1976).

Luh and Woo (1950) supposed that *I.pomerantzevi* is possibly a synonym of *Ixodesangustus*; Filippova (1977) considered it as valid and in the last list of valid tick species names, it is also considered valid (Guglielmone et al. 2020).

*Ixodespomerantzevi* is a nidicolous tick species, an ectoparasite of rodents, hedgehogs, and shrews (Filippova 1977).

The type specimen of *I.pomerantzevi* is deposited at the Zoological Institute of the Russian Academy of Sciences and include the holotype: female; [Russia], DVK [Primorskii Terr.], Suputinskii [Komarovskii or Ussurisky] Nature Reserve, from *Myodesrufocanus*, 9–13.VI.1939, coll. B.I. Pomerantsev; AL I502. Description – Filippova 1977: 128–132 (female, male - unknown, nymph, larva) (Filippova 2008).

##### ﻿ Ixodes stromi

Taxon classificationAnimaliaIxodidaIxodidae

Filippova, 1957

FEC52874-4C43-5429-9030-31C868A95539

Ixodes stromi Filippova, 1957: 864.

###### Recorded hosts.

**Mammalia**: *Alticolaargentatus* (Severtzov) (silver mountain vole), *Apodemusagrarius* (striped field mouse), *Craseomysrufocanus* (grey red-backed vole), *Crocidura* sp. (shrew), *Lasiopodomysgregalis* (narrow-headed vole), *Microtusarvalis* (common vole), *Mustela* sp. (weasel), *Myodescentralis* (Miller) (Tien Shan red-backed vole), *Nothocricetulusmigratorius* (grey dwarf hamster), *Ochotonamacrotis* (Günther) (large-eared pika), *Rattuspyctoris* (Turkestan rat) (Filippova 1977).

###### Recorded locations

**Figure 20.**
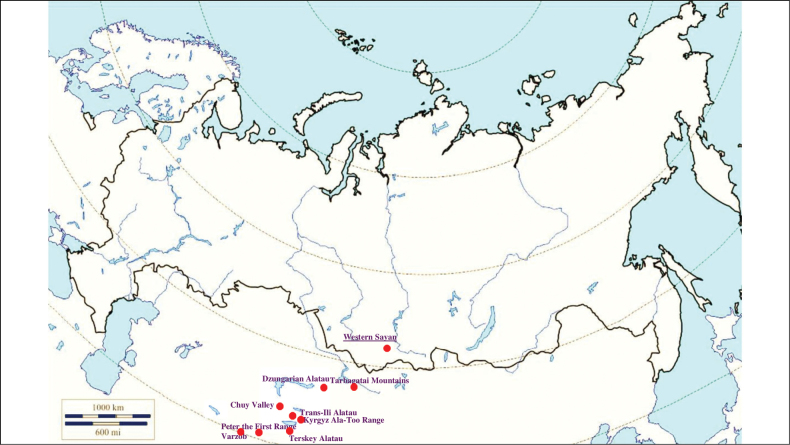
Map of Russia and neighboring countries showing the locations where *Ixodesstromi* was reported.

**(Fig. 20). Russia**: Western Sayan (Arumova and Dineva 1973). **Kazakhstan**: Tarbagatai Mountains (Afanas’eva 1959), Dzungarian Alatau (Ushakova and Fedosenko 1963; Ushakova et al. 1976), Trans-Ili Alatau (Ushakova and Fedosenko 1963). **Kyrgyzstan**: Kyrgyz Ala-Too Range (Fedorova 2012b), Terskey Alatau (Fedorova 2012b), Chuy Valley – found in 1966 (Grebenyuk 1966), was not found in the same territories in 2018 (Fedorova 2021). **Tajikistan**: Peter the First Range (Filippova 1977), Varzob gorge (Sosnina 1954 – here *I.stromi* was incorrectly identified as *I.trianguliceps* because the new species was described by Filippova in 1957b).

###### Ecology and other information.

*Ixodesstromi* is a tick species only indigenous to southern Siberia in Russia (Tsapko 2020). The main part of its distribution spans in Kazakhstan and Middle Asia. In all territories of its range, it is confined to the forest-meadow and forest-steppe belt of medium-altitude mountains, to stony and rocky habitats, which are insolated and, therefore, have a warmer microclimate (Filippova 1967).

This species is nidicolous and uses rodents, shrews, and small carnivores as hosts at all stages. It is considered a rare species reaching small individual number (Filippova 1977).

The type specimens of *I.stromi* are known from Kyrgyzstan and stored at the Zoological Institute of the Russian Academy of Sciences: the lectotype: the nymph; Kyrgyzstan, Tien Shan, Kungei Ala Tau Mt. Range, Ch-Aksu Canyon, talus, from *Clethrionomysfrater* (synonym of *Myodescentralis*), 11.VIII.1953, coll. N.А. Filippova; AL I78. The paralectotypes: 6 larvae; FBM I586, I876; 6 larvae; FBM I873, I875. Description – Filippova 1977: 122–127 (female, nymph, larva; male unknown) (Filippova 2008).

#### Subgenus ﻿Monoixodes Emelyanova & Kozlovskaya, 1967: 489.

##### ﻿ Ixodes maslovi

Taxon classificationAnimaliaIxodidaIxodidae

Emelyanova & Kozlovskaya, 1967

AFF195C4-A5A3-5CB7-B567-07E9184B0408

Ixodes maslovi Emelyanova & Kozlovskaya, 1967: 489.

###### Recorded hosts.

To date hosts of this tick species are unknown (Guglielmone et al. 2014).

###### Recorded locations

**Figure 21.**
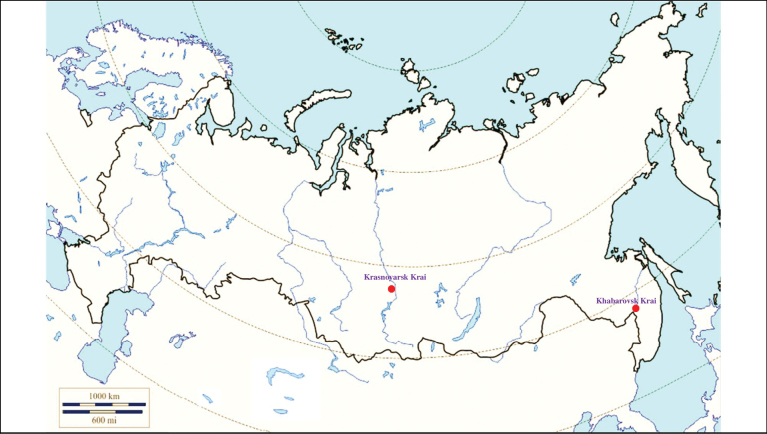
Map of Russia and neighboring countries showing the locations where *Ixodesmaslovi* was reported.

**(Fig. 21). Russia**: Khabarovsk Krai – Khekhtsir Range and the rural locality Vyatskoye (Emelyanova and Kozlovskaya 1967); Krasnoyarsk Krai – Kozulsky District, the village Bolshoy Kemchug (Voltsyt 1997).

###### Ecology and other information.

*Ixodesmaslovi* is an almost unstudied tick known and described from two findings of its male and female (Emelyanova and Kozlovskaya 1967), as well as the nymph (Voltsyt 1997).

Camicas et al. (1998) and Kolonin (2009) regard *I.maslovi* as an abnormal form of *I.persulcatus* although Filippova (1977) and Guglielmone et al. (2020) consider *I.maslovi* a valid taxon.

The type specimens are deposited at the Zoological Institute of the Russian Academy of Sciences – the holotype: male; [Russia], environs of Khabarovsk, Khekhtsir Mt. Range, 12.VI.1964, collected from vegetation by O.L. Kozlovskaya; FBM I1412; the paratype: female; FBM I1413. Description – Filippova 1977: 248–251 (female, male); Voltsit 1997: 265–268 (nymph; larva unknown) (Filippova 2008).

#### ﻿Subgenus ﻿Pholeoixodes Schulze, 1942: 630.

##### ﻿ Ixodes arboricola

Taxon classificationAnimaliaIxodidaIxodidae

Schulze & Schlottke, 1929

0838E124-7D1F-55BD-A058-4C7A185AE150

Ixodes arboricola Schulze & Schlottke: Morel and Pérez 1973: 275.
Ixodes arboricola muscicapae Schulze, 1930: 3; Haarløv 1962: 425.
Ixodes strigicola Schulze & Schlottke: Haarløv 1962: 425.
Ixodes dryadis Schulze & Schlottke: Haarløv 1962: 425.
Ixodes passericola Schulze: Haarløv 1962: 425.
Ixodes arboricola bogatschevi Kirshenblat, 1936: 93; Haarløv 1962: 425.
Ixodes lagodechiensis Dzhaparidze, 1950: 117; Kolonin 1981: 84.

###### Recorded hosts.

**Aves**: *Accipitergentilis* (Linnaeus) (northern goshawk), *Acrocephalusscirpaceus* (Hermann) (Eurasian reed warbler), *Aegithaloscaudatus* (Linnaeus) (long-tailed tit), *Aegoliusfunereus* (Linnaeus) (boreal owl), *Athenenoctua* (little owl), *Certhiabrachydactyla* Brehm (short-toed treecreeper), *Certhiafamiliaris* Linnaeus (Eurasian treecreeper), *Chlorischloris* (European greenfinch), *Coloeusmonedula* (Linnaeus) (western jackdaw), *Columbapalumbus* Linnaeus (common wood pigeon), *Coraciasgarrulus* Linnaeus (European roller), *Corvusfrugilegus* Linnaeus (rook), *Currucacommunis* (common whitethroat), *Cyanistescaeruleus* (Linnaeus) (Eurasian blue tit), *Dendrocoposmajor* (great spotted woodpecker), *Emberizacitrinella* (yellowhammer), *Erithacusrubecula* (European robin), *Falcoperegrinus* Tunstall (peregrine falcon), *Falcotinnunculus* Linnaeus (common kestrel), *Ficedulaalbicollis* (Temminck) (collared flycatcher), *Ficedulahypoleuca* (European pied flycatcher), *Garrulusglandarius* (Eurasian jay), *Glaucidiumpasserinum* (Linnaeus) (Eurasian pygmy owl), *Hirundorustica* Linnaeus (barn swallow), *Lophophanescristatus* (Linnaeus) (crested tit), *Motacillaalba* (white wagtail), *Muscicapastriata* (Pallas) (spotted flycatcher), *Parusmajor* (great tit), *Passerdomesticus* (house sparrow), *Passermontanus* (Eurasian tree sparrow), *Periparusater* (Linnaeus) (coal tit), *Phoenicurusochruros* (Gmelin) (black redstart), *Phoenicurusphoenicurus* (common redstart), *Phylloscopustrochilus* (Linnaeus) (willow warbler), *Picuscanus* Gmelin (grey-headed woodpecker), *Poecilemontanus* (Conrad von Baldenstein) (willow tit), *Poecilepalustris* (Linnaeus) (marsh tit), *Pyrrhulapyrrhula* (Linnaeus) (Eurasian bullfinch), *Remizpendulinus* (Linnaeus) (Eurasian penduline tit), *Ripariariparia* (Linnaeus) (sand martin), *Serinusserinus* (Linnaeus) (European serin), *Sittaeuropaea* (Eurasian nuthatch) (Filippova 1977; Keve et al. 2022), western rock nuthatch *Sittaneumayer* Michahelles (Ogandzhanyan 1984), *Spinusspinus* (Linnaeus) (Eurasian siskin), *Strixaluco* Linnaeus (tawny owl), *Sturnusvulgaris* (common starling), *Troglodytestroglodytes* (Linnaeus) (Eurasian wren), *Turdusmerula* (common blackbird), *Turdusphilomelos* (song thrush), *Tytoalba* (Scopoli) (barn owl), *Upupaepops* Linnaeus (Eurasian hoopoe) (Filippova 1977; Keve et al. 2022).

###### Recorded locations

**Figure 22.**
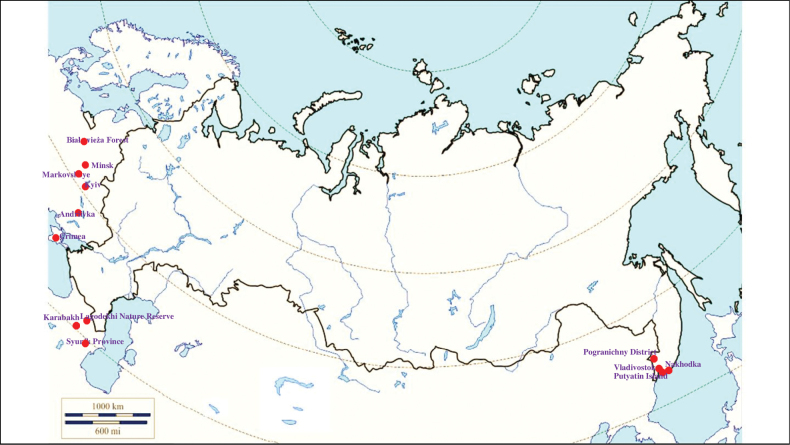
Map of Russia and neighboring countries showing the locations where *Ixodesarboricola* was reported.

**(Fig. 22). Russia**: Southern Primorskyi Krai (Pogranichny District, Vladivostok, Nakhodka, Putyatin Island) (Emelyanova and Gordeeva 1969; Emelyanova 1972; Bolotin 2000). **Ukraine**: outskirts of Kyiv (Nebogatkin 2014), Dnipropetrovsk Oblast (the rural locality Andriivka), Crimea (Olenivka and Alushta) (Filippova 1977). **Belarus**: Białowieża Forest, Minsk, Gomel Oblast (the village Markovskoye) (Gembetsky 1966, 1972). **Armenia**: Syunik Province (former Goris Province) (Ogandzhanyan 1984). **Azerbaijan**: Karabakh (Kirschenblatt 1936). **Georgia**: Lagodekhi Nature Reserve (Djaparidze 1960). **Kyrgyzstan** (Fedorova 2012a).

###### Ecology and other information.

*Ixodesarboricola* is an endophilic parasite, mainly of birds from ecological groups nesting in tree holes and nest boxes and also even in ground burrows (Filippova 1977). Also, certain cases of this species infesting bats in tree holes have been recorded (Arthur 1963).

The interesting feature of its distribution is the disjunctivity, which is confirmed by the discovery of this species in the areas quite distant from each other – western and central Europe, North Africa, Transcaucasia, western Asia, and the Far East in Russia (Estrada-Peña et al. 2018) and China (Chen et al. 2010).

##### ﻿ Ixodes cornutus

Taxon classificationAnimaliaIxodidaIxodidae

Lotozky, 1956

CA745B71-5DDD-570F-B9A8-2A11F2E5092B

Ixodes cornutus Lototsky, 1956: 27.
Ixodes rugicollis Schulze & Schlottke: Morel and Aubert 1975: 99.

###### Recorded hosts.

**Mammalia**: *Mustelaerminea* Linnaeus (stoat) (Filippova 1977).

###### Recorded locations

**Figure 23.**
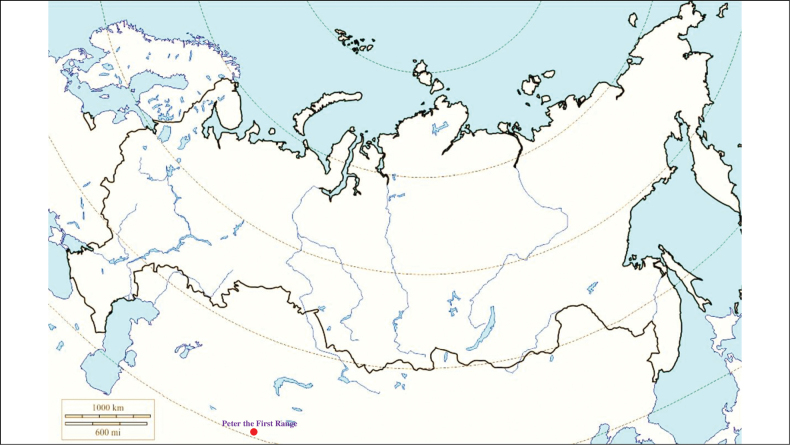
Map of Russia and neighboring countries showing the locations where *Ixodescornutus* was reported.

**(Fig. 23). Tajikistan**: Peter the First Range, the source of the Divansu River, close to the Oshanin glacier (Filippova 1977).

###### Ecology and other information.

*Ixodescornutus* is a species described from two identical females (Lotozky 1956) that were found in Tajikistan, in the eastern part of Peter the First Range, by the source of the Divansu River (the basin of the Surkhob River), near the Oshanin glacier, on a stoat.

The type specimen of *I.cornutus* is deposited at the Zoological Institute of the Russian Academy of Sciences (Lotozky 1956: 27). Lectotype: female; 38 [Tajikistan, the Peter the First Mt. Range], the source of the Divansu River, the ancient moraine of the Oshanin glacier, *Mustelaerminea*, ad.; male; 4.VII.1954; AL I845. Description – Filippova 1977: 178 (female; male, nymph, larva unknown) (Filippova 2008).

##### ﻿ Ixodes crenulatus

Taxon classificationAnimaliaIxodidaIxodidae

Koch, 1844

A9937B17-11CA-57E7-A70D-A2D3DF8496EC

Ixodes crenulatus Koch, 1844c: 39; Morel and Pérez 1973: 275.

###### Note.

Tick names are used sensu Guglielmone et al. (2014) in this review. Thus, this species is not synonymous with *I.canisuga* Johnston as suggested by Filippova (1977) based on their morphological similarities and because the latter is not known to occur in Russia. *Ixodescrenulatus* was erroneously synonymized with *I.kaiseri* Arthur (Sonenshine et al. 1969), as clarified later (Filippova and Uspenskaya 1973).

###### Recorded hosts.

**Mammalia**: *Allactagamajor* (Kerr) (great jerboa), *Allactagasibirica* (Forster) (Mongolian five-toed jerboa), *Allocricetuluseversmanni* (Brandt) (Eversmann’s hamster), *Apodemussylvaticus* (wood mouse), *Canisaureus* Linnaeus (golden jackal), *Canisfamiliaris* (domestic dog), *Canislupus* Linnaeus (gray wolf), *Cricetulusbarabensis* (Pallas) (Chinese striped hamster), *Ellobiustalpinus* (northern mole vole), *Erinaceuseuropaeus* (European hedgehog), *Feliscatus* Linnaeus (domestic cat), *Felislybica* Forster (African wildcat), *Hemiechinusauratus* (long-eared hedgehog), *Homosapiens* Linnaeus (human), *Lasiopodomysgregalis* (narrow-headed vole), *Lepustolai* (tolai hare), *Marmotabaibacina* (gray marmot), *Marmotabobak* (bobak marmot), *Marmotacaudata* (Geoffroy) (long-tailed marmot), *Marmotakastschenkoi* Stroganov and Yudin (forest-steppe marmot), *Marmotamenzbieri* (Kashkarov) (Menzbier’s marmot), *Marmotasibirica* (Tarbagan marmot), *Melesmeles* (Eurasian badger), *Microtusarvalis* (common vole), *Mustelaeversmanii* (steppe polecat), *Mustelanivalis* (least weasel), *Myodesglareolus* (bank vole), *Myospalaxmyospalax* (Siberian zokor), *Nothocricetulusmigratorius* (grey dwarf hamster), *Nyctereutesprocyonoides* (Gray) (common raccoon dog), *Ochotonadauurica* (Pallas) (Daurian pika), *Ochotonapallasi* (Gray) (Pallas’s pika), *Otocolobusmanul* (Pallas) (Pallas’s cat), *Ovisaries* (domestic sheep), *Phodopussungorus* (Pallas) (winter white dwarf hamster), *Procyonlotor* (Linnaeus) (raccoon), *Spermophilusdauricus* Brandt (Daurian ground squirrel), *Spermophiluspygmaeus* (little ground squirrel), *Spermophilusrelictus* (Kashkarov) (relict ground squirrel), *Spermophilussuslicus* (speckled ground squirrel), *Vulpescorsac* (corsac fox), *Vulpesvulpes* (red fox) (Filippova 1977; Litvinov and Sapegina 2003; Kalyagin et al. 2005, 2008).

**Aves**: *Emberizacia* Linnaeus (rock bunting), *Oenantheisabellina* (isabelline wheatear) (Filippova 1977).

###### Recorded locations

**Figure 24.**
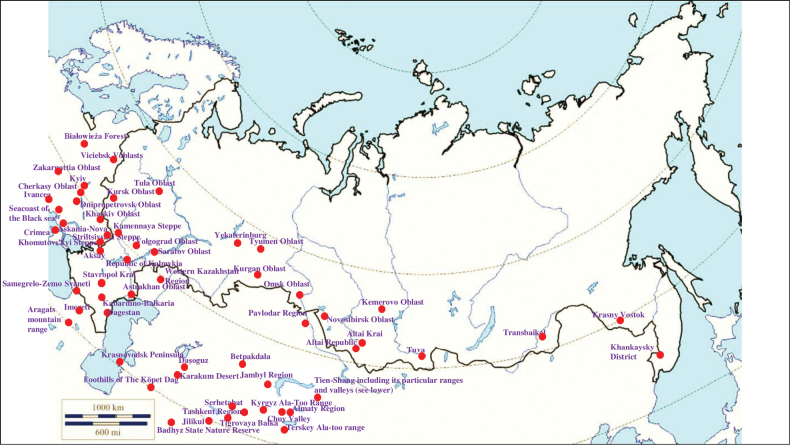
Map of Russia and neighboring countries showing the locations where *Ixodescrenulatus* was reported.

**(Fig. 24). Russia**: Tula Oblast (Myasnikov and Katelina 1964), Kursk Oblast (Lgovsky District), Voronezh Oblast (Kamennaya Steppe Nature reserve), Rostov Oblast (Aksay), Republic of Kalmykia (Derbetovsky District, Sarpinsky District), Volgograd Oblast (Gorodishchensky, Derbetovsky and Sarpinsky District) (Denisov 2010, 2019), Kabardino-Balkaria (tract Khaimasha) (Bittirova et al. 2019), Dagestan (Aliev et al. 2007), Astrakhan Oblast, Stavropol Krai (Filippova 1977), Saratov Oblast (Turtseva 2007; Denisov 2010, 2019; Porshakov et al. 2020), Yekaterinburg (Milintsevich et al. 2016), Tyumen Oblast (Glazunov and Zotova 2014), Kurgan Oblast (Starikov and Starikova 2021), Novosibirsk Oblast (Suzunsky, Karasuksky and Maslyaninsky District) (Davydova and Lukin 1969), Omsk Oblast (Tarasevich et al. 1971), Kemerovo Oblast (Kalyagin et al. 2005, 2008; Kovalevsky et al. 2018); Altai Krai (Oberth et al. 2015) (Sovetsky District, the village Kokshi) (Filippova 1977), Altai Republic (Shebalinsky District, the village Cherga) (Litvinov and Sapegina 2003), Tuva (Glazunov and Zotova 2014; Filippova 1977), Transbaikal (villages Borgoy, Kyakhta, Selenge and Borzinsky District) (Filippova 1977); Amur Oblast (village Krasny Vostok), Southern Outer Manchuria (Khankaysky District (Kolonin 1986; Bolotin 2000). **Ukraine**: outskirts of Kyiv (Akimov and Nebogatkin 2016), Zakarpattia Oblast and Western Ukraine in general (Podobivskyi and Fedonyuk 2017), Cherkasy Oblast, Dnipropetrovsk Oblast, Askania-Nova Nature Reserve, Striltsivskyi Steppe Nature Reserve, Kharkiv Oblast (Tokarsky and Zorya 2007), Lugansk Oblast (Kuznetsov and Bondarev 2007) (including Khomutovs’kyi Steppe) (Filippova 1977), the north-western sea coast of the Black Sea (Rusev 2009), Crimea (Evstafiev 2017) – plain and mountainous lands (Filippova 1977). **Belarus**: Viciebsk Voblasts (Subbotina and Osmolovsky 2022), Białowieża Forest (Filippova 1977), considered rare (Bychkova et al. 2015). **Moldova**: Lozova, Ivancea, Leova, reedbeds of the low Dniester and Pruth (Filippova 1977; Uspenskaya et al. 2006). **Georgia**: Samegrelo-Zemo Svaneti, Imereti (Sukhiashvili et al. 2020). **Armenia**: Aragats mountain range (Dilbaryan and Poghosyan 2018). **Kazakhstan**: through the whole territory of Kazakhstan (Filippova 1977) and plus recent findings in the next regions: West Kazakhstan Region (Tanitovsky and Maikanov 2018), Almaty Region (Bibikov and Bibikova 2010), Pavlodar Region (Amirova et al. 1989), the north of Betpak-Dala (Rapoport et al. 2017), Jambyl Region (Kyrgyz Ala-Too Range, Talas Alatau) (Sarsenbaeva et al. 2016). **Kyrgyzstan**: Tian Shan in general (Abdikarimov et al. 2018) and its certain ranges and valleys including Kyrgyz Ala-Too Range (Akyshova et al. 2022) and Terskey Ala-too Range (Fedorova 2012b); Chuy Valley (Fedorova 2021). **Turkmenistan**: Krasnovodsk Peninsula, Daşoguz, the foothills of The Köpet Dag, Badhyz State Nature Reserve, Karakum Desert (Kochkareva et al. 1971), Serhetabat (former Kushka) (Filippova 1977). **Uzbekistan**: Tashkent Region (Muratbekov 1954). **Tajikistan**: outskirts of the rural locality Jilikul (Filippova 1977), Tigrovaya Balka (Manilova and Shakhmatov 2008).

###### Ecology and other information.

*Ixodescrenulatus* is among the tick species that have the most extensive ranges comparing to other representatives of its family within Russia (Tsapko 2020).

It is a typical nidicolous parasite of mammals and in the Asian part of its range as the main hosts it uses species of marmots of the genus *Marmota* (with a predominance of gray marmot) and such representatives of predatory mammals as badgers, steppe polecats, red and corsac foxes. The composition of the main host spectrum from different orders (rodents and predatory mammals) finds an explanation in close connections of topical and trophic relationships of marmots and predators. All of them have burrows of medium diameter, complex design, with a nesting chamber, remote from the entrance, which provides the stability of the microclimate, where ticks find suitable conditions. The above species of carnivores often use the burrows of their prey, marmots, and small carnivores, facilitating the exchange of ticks not only between individual burrows, but also between remote host settlements (Filippova 2011).

This tick species is considered rare, for example, only few findings were mentioned in the Astrakhan Oblast (Zimina et al. 1965; Zimina et al. 1996) and Saratov Oblast (Denisov 2019). In most of the recognized range, *I.crenulatus* coexists with the closely related *I.kaiseri*. These species not only inhabit the same territory and the same biotopes but can also parasitize one host individual at the same time (Tsapko 2017). Therefore, it is necessary to consider that accurate identification of these species is required and there is always a chance of their misidentification.

According to some suggestions (Emelyanova 1979), *I.crenulatus* is probably a species group, or at least has remarkable intraspecific variations involving morphotypes (Filippova and Panova 2000).

##### ﻿ Ixodes hexagonus

Taxon classificationAnimaliaIxodidaIxodidae

Leach, 1815

671A2F1E-AF9C-5B24-9023-C6B9BAACE49F

Ixodes hexagonus Leach, 1815: 397; Morel and Pérez 1973: 275.
Ixodes autumnalis Leach: Neumann 1911: 17.
Ixodes erinacei Audouin: Neumann 1911: 17.
Ixodes auricularis Robineau-Desvoidy: Morel and Pérez 1973: 275.
Ixodes sexpunctatus Koch: Neumann 1911: 17.
Ixodes vulpis Pagenstecher: Neumann 1911: 17.
Ixodes erinaceus Audouin: Neumann 1911: 17.

###### Recorded hosts.

**Mammalia**: *Bostaurus* Linnaeus (cattle), *Canisfamiliaris* (domestic dog), *Erinaceuseuropaeus* (European hedgehog), *Feliscatus* (domestic cat), *Lutralutra* (Linnaeus) (Eurasian otter), *Melesmeles* (Eurasian badger), *Mustelaerminea* (stoat), *Mustelanivalis* (least weasel), *Mustelaputorius* Linnaeus (European polecat), *Oryctolaguscuniculus* (Linnaeus) (European rabbit), *Ovisaries* (sheep), *Rattusnorvegicus* (brown rat), *Vulpesvulpes* (red fox) (Filippova 1977).

**Aves**: *Turdusmerula* (common blackbird) (Filippova 1977).

###### Recorded locations

**Figure 25.**
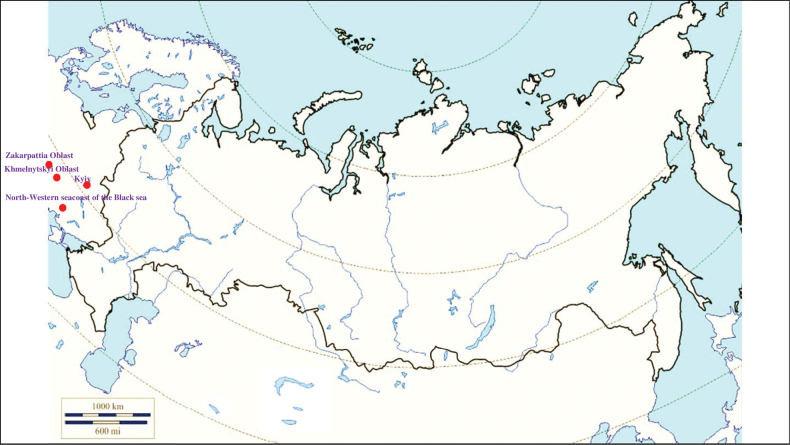
Map of Russia and neighboring countries showing the locations where *Ixodeshexagonus* was reported.

**(Fig. 25). Ukraine**: outskirts of Kyiv, Khmelnytskyi Oblast (Levytska et al. 2021), the North-Western seacoast of the Black Sea (Rusev 2009), Zakarpattia Oblast (the rural locality Malyi Berezny) (Filippova 1961).

###### Ecology and other information.

*Ixodeshexagonus* is a typical nidicolous parasite of carnivores and hedgehogs. It has certain morphological similarities to *I.crenulatus* and *I.kaiseri* and has common sympatric zones with this species along its range (Filippova 1999). Ukraine is the only country of the former Soviet Union, on the territory of which this European species is present in the tick fauna (Filippova 1977). In general *I.hexagonus* was detected quite rarely in Ukraine, and almost always in the west of Ukraine and mainly from hedgehogs (Kolonin 2009). Akimov and Nebogatkin (2016) assumed that it can be found in the vicinity of Kyiv, and eventually it was confirmed by Levytska et al. (2021). Rare occasional human bites have been recorded (Rosický and Weiser 1952; Arthur 1963; Bursali et al. 2012).

##### ﻿ Ixodes kaiseri

Taxon classificationAnimaliaIxodidaIxodidae

Arthur, 1957

21C70593-4580-5234-8551-91331C2F7F48

Ixodes kaiseri Arthur, 1957: 578; Morel and Aubert 1975: 99.
Ixodes bakonyensis Babos: Morel and Aubert 1975: 99.
Ixodes vulpinus Babos: Morel and Aubert 1975: 99.

###### Recorded hosts.

**Mammalia**: *Canisfamiliaris* (domestic dog), *Erinaceusconcolor* Martin (southern white-breasted hedgehog), *Erinaceuseuropaeus* (European hedgehog), *Erinaceusroumanicus* Barrett-Hamilton (northern white-breasted hedgehog), *Felischaus* Schreber (jungle cat), *Felislybica* (African wildcat), *Hyaenahyaena* (Linnaeus) (striped hyena), *Hystrixindica* Kerr (Indian crested porcupine), *Lepuseuropaeus* (brown hare), *Mustelaeversmanii* (steppe polecat), *Melesmeles* (Eurasian badger), *Nyctereutesprocyonoides* (common raccoon dog), *Vulpescorsac* (corsac fox), *Vulpesvulpes* (red fox) (Filippova 1977; Tsapko 2017).

###### Recorded locations

**Figure 26.**
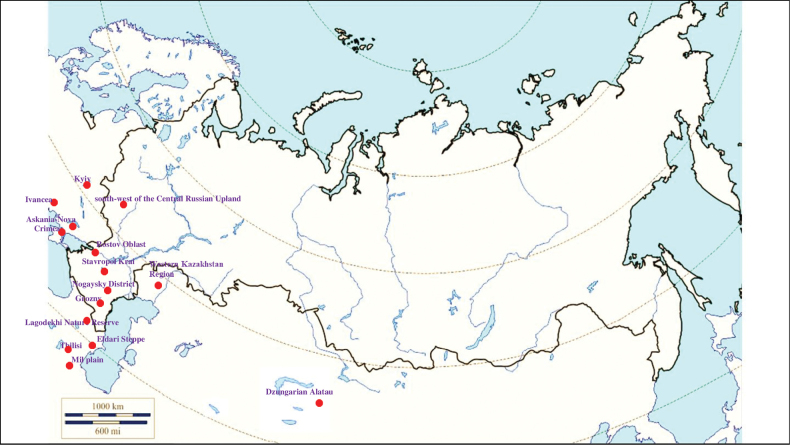
Map of Russia and neighboring countries showing the locations where *Ixodeskaiseri* was reported.

**(Fig. 26). Russia**: southwestern peripheries of the Central Russian Upland and also Rostov Oblast (Khametova et al. 2018) and Stavropol Krai (Tsapko 2017) and the North Caucasus – the outskirts of Grozny (Chechnya) (Filippova 1977, 1999; Tsapko 2017, 2020) and Nogaysky District (Dagestan) (Tsapko 2017). **Ukraine**: outskirts of Kyiv (Akimov and Nebogatkin 2016) and the south of Ukraine, in particular Askania-Nova Nature Reserve (Filippova 1977), the North-Western seacoast of the Black Sea (Matyukhin 2017), Crimea (Filippova 1977). **Moldova**: Lozova, Ivancea, Doibani, Leova, Etulia, reedbeds of the low Dniester and Pruth (Filippova 1977; Uspenskaya et al. 2006). **Georgia**: the outskirts of Tbilisi, Lagodekhi Nature Reserve (Filippova 1977), Eldari Steppe (Tsapko 2017). **Armenia**: Gegharkunik Province (rural locality Geghamashen) (Tsapko 2017), Aragats mountain range (Dilbaryan and Poghosyan 2018). **Azerbaijan**: Mil plain (Filippova and Uspenskaya 1973), Beylagan District, Zangilan District, Aghjabadi District, Martuni Province, Shaki District (Tsapko 2017). **Kazakhstan**: West Kazakhstan Region, Dzungarian Alatau – outskirts of the rural locality Topolëvka and the Koksu district (Ushakova et al. 1976; Filippova 1999). **Kyrgyzstan** (Fedorova 2012b).

###### Ecology and other information.

*Ixodeskaiseri* is a typical nidicolous parasite of carnivores and also hedgehogs and porcupines which is morphologically very similar to *I.crenulatus* and has common sympatric zones with this species along its range (Filippova 1999). Its range itself is patchy and disjunctive areas of sympatry for both of these species are found in southeastern Europe - Romania, Moldova, Ukraine, including the Crimean Peninsula as well as in Russia - the southwestern extremities of the Central Russian Upland and the Northern Caucasus; then after a long gap - in Western Kazakhstan and again after a big gap - in the Dzungarian Alatau (Filippova 1999). Judging by literature, it is also known from Egypt and Israel (Arthur 1957, 1960, 1965). As Filippova and Uspenskaya (1973) assumed, its distribution in the Middle East and also other parts of Asia can be wider than it is known at present, which was already confirmed by the findings of this tick species in Turkey (Orkun and Karaer 2018) and Xinjiang in China (Zhao et al. 2019) near the border with Kazakhstan and the Dzungarian Alatau and hence, it is possible that the sympatry of these two species is more widespread. This is also supported by literature data, because until the 1970s in the territory of the former USSR *I.kaiseri* was not differentiated from *I.hexagonus* and *I.crenulatus* (Emelyanova 1979; Filippova 1999).

These species not only inhabit the same territory and inhabit the same biotopes in some places but can also parasitize one host individual at the same time (Tsapko 2017). It is also important to note that according to Filippova’s opinion (1999), the territorial signs of the ranges of these two species, their biotope and host-parasite relationships indicate that the range of *I.crenulatus* (which is predominantly connected with marmots and their burrows in steppe and forest-steppe zones) over the most part of its distribution has a Central Asian origin, while the range of *I.kaiseri* (mainly the parasite of carnivores and occurring in their burrows) is supposedly of European origin.

##### ﻿ Ixodes lividus

Taxon classificationAnimaliaIxodidaIxodidae

Koch, 1844

CBB38AA7-685A-56A0-90C8-8ADA8A259FC6

Ixodes lividus Koch, 1844: 234; Morel and Pérez 1973: 275.
Ixodes bavaricus Schulze & Schlottke, 1929: 95.
Ixodes plumbeus bavaricus Schulze & Schlottke: Morel and Pérez 1973: 275.
Ixodes plumbeus obotriticus Schulze & Schlottke: Morel and Pérez 1973: 275.
Ixodes (Pholeoixodes) hirundinicola Schulze: Kolonin 1981: 19.

###### Recorded hosts.

**Aves**: *Alaudaarvensis* (Eurasian skylark), *Delichonurbicum* (Linnaeus) (common house martin), *Meropsapiaster* Linnaeus (European bee-eater), *Passerdomesticus* (house sparrow), *Passermontanus* (Eurasian tree sparrow), *Ripariadiluta* (Sharpe & Wyatt) (pale martin), *Ripariariparia* (sand martin) (Filippova 1977; Tagiltsev et al. 1984; Rusev 2009; Bolshakova et al. 2019; Kovalevsky et al. 2019).

**Mammalia**: *Musmusculus* (house mouse) (Filippova 1977).

###### Recorded locations

**Figure 27.**
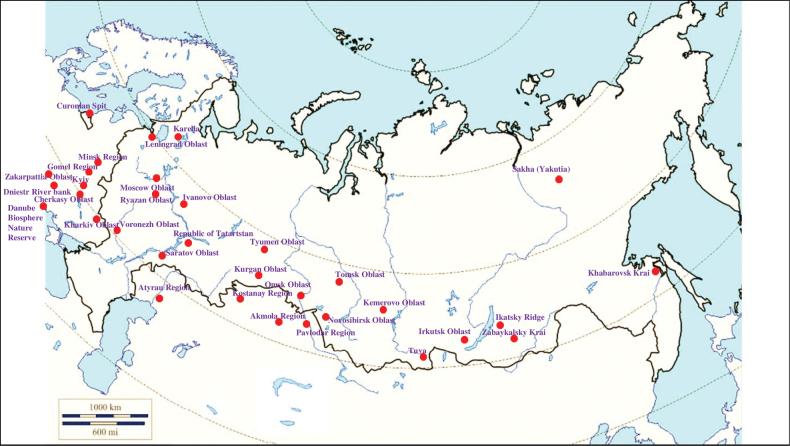
Map of Russia and neighboring countries showing the locations where *Ixodeslividus* was reported.

**(Fig. 27). Russia**: Curonian Spit (Kaliningrad Oblast) (Filippova 1961), Karelia (Bobrovskikh 1979), Leningrad Oblast, (Zolotov and Buker 1976), Moscow Oblast (Glashchinskaya-Babenko 1956), Ryazan Oblast (Filippova 1977), Ivanovo Oblast (Mayorova 2004), Saratov Oblast (Korneev et al. 2018), Kuybyshev Reservoir (Republic of Tatartstan) (Lvov et al. 2014), Voronezh Oblast (Gaponov and Tewelde 2021), Tymen Oblast (Starikov et al. 2017b), Kurgan Oblast (Starikov and Starikova 2021), Omsk Oblast (Tagiltsev et al. 1984; Yakimenko et al. 1991), Tomsk Oblast, Kemerovo Oblast (Kovalevsky et al. 2019), Novosibirsk Oblast (Yakimenko et al. 2013), Irkutsk Oblast (Danchinova et al. 2007), Ikatsky Ridge (Republic of Buryatia), Zabaykalsky Krai (Emelyanova et al. 1963), Republic of Tuva (Kholodilov et al. 2019), Sakha (Yakutia) (Shadrina et al. 2011), Khabarovsk Krai (Volkov and Chernykh 1977). **Ukraine**: Zakarpattia Oblast, Kaniv Nature Reserve (Cherkasy Oblast), (Emchuk 1960), Danube Biosphere Nature Reserve (Odesa Oblast) (Emchuk 1960; Didyk 2013), Kyiv (Akimov and Nebogatkin 2002), delta of the Dniestr River (Rusev 2009). **Belarus**: Gomel Region, Minsk Region (Gembetsky 1972). **Moldova**: banks of the Dniestr River (Movila et al. 2008). **Kazakhstan**: Atyrau Region (Pomerantsev 1950; Levit 1957), Kostanay Region (Makhmetov 1961), Pavlodar Region (Amirova et al. 1989), Akmola Region (Filippova 1961; Ushakova 1962).

###### Ecology and other information.

*Ixodeslividus* is a specific nidicolous ectoparasite of the sand martin, *Ripariariparia*. Also, it has been collected from birds and house mice which occasionally could visit sand martin’s nests such as house sparrows and common house martins.

Due to the wide distribution of its main host, this tick species also occurs in a vast geographical range and can be characterized by having a trans-Palearctic distribution. The locations of findings in Russia and the neighboring countries listed above reflect the general pattern of its distribution on a map so we can suppose that this tick can be found in the north of the Palearctic almost everywhere in habitats of the sand martin.

##### ﻿ Ixodes prokopjevi

Taxon classificationAnimaliaIxodidaIxodidae

(Emel´yanova, 1979)

B423D677-7261-56E3-9617-3922FD126FDC

Pholeoixodes prokopjevi Emel’yanova, 1979: 14.

###### Recorded hosts.

**Mammalia**: Daurian hedgehog *Mesechinusdauuricus* (Sundevall) (Emelyanova 1979).

###### Recorded locations

**Figure 28.**
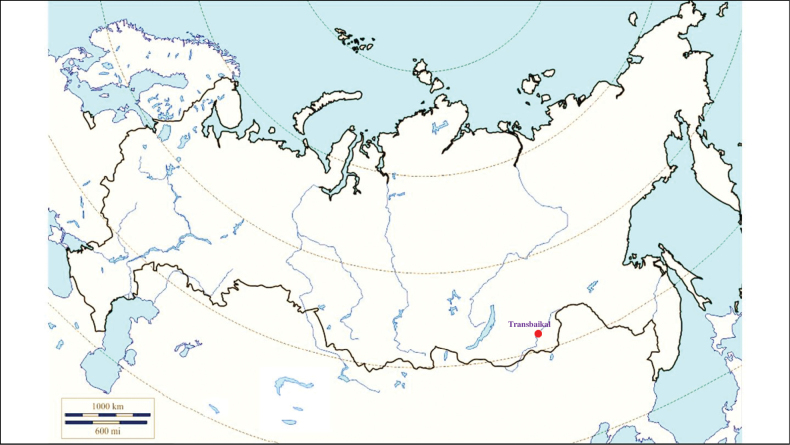
Map of Russia and neighboring countries showing the locations where *Ixodesprokopjevi* was reported.

**(Fig. 28). Russia**: Transbaikal (Emelyanova 1979).

###### Ecology and other information.

*Ixodesprokopjevi* is an extremely poorly studied tick species initially described based on the male holotype from steppes of north-eastern Mongolia; its paratypes, larvae and nymphs, are noted as originating from the outskirts of the lake Baruun Shavart Nuur in Eastern Mongolia, as well as females and nymphs from the south-eastern Transbaikal without any indications of certain points of findings (Emelyanova 1979).

Kolonin (2009) states that this species should be considered a synonym of *I.crenulatus* but Guglielmone et al. (2010, 2014) recognize it as a valid species.

The Daurian hedgehog was recorded as a host, but we can assume that carnivores, lagomorphs, and rodents are also hosts of this tick species, as in case of *I.crenulatus*, another representative of the subgenus ﻿Pholeoixodes and the most similar species to this tick. The distribution area and ecology of *I.prokopjevi*, as well host-parasite relationships and their role in transmission of vector-borne infections remain unknown.

##### ﻿ Ixodes subterraneus

Taxon classificationAnimaliaIxodidaIxodidae

Filippova, 1961

8183E088-2C89-5E9E-8DFC-0C3809BBC08D

Ixodes subterranus Filippova, 1961: 226. Morel and Pérez 1973: 275.
Pholeoixodes arboricola koshkinae Emel’yanova: Kolonin 1981: 20.
Pholeoixodes arboricola deserta Emel’yanova: Kolonin 1981: 20.

###### Recorded hosts.

**Aves**: *Athenenoctua* (little owl), *Cardueliscarduelis* (European goldfinch), *Coraciasgarrulus* (European roller), *Coturnixcoturnix* (common quail), *Galeridacristata* (crested lark), *Falconaumanni* Fleischer (lesser kestrel), *Oenanthehispanica* (western black-eared wheatear), *Oenantheisabellina* (isabelline wheatear), *Oenantheoenanthe* (northern wheatear), *Parusmajor* (great tit), *Passerammodendri* Gould (saxaul sparrow), *Passerdomesticus* (house sparrow), *Passermontanus* (Eurasian tree sparrow), *Pastorroseus* (rosy starling), *Petroniapetronia* (rock sparrow), *Picapica* (Eurasian magpie), *Sturnusvulgaris* (common starling), *Turdusruficollis* (red-throated thrush) (Filippova 1977).

###### Recorded locations

**Figure 29.**
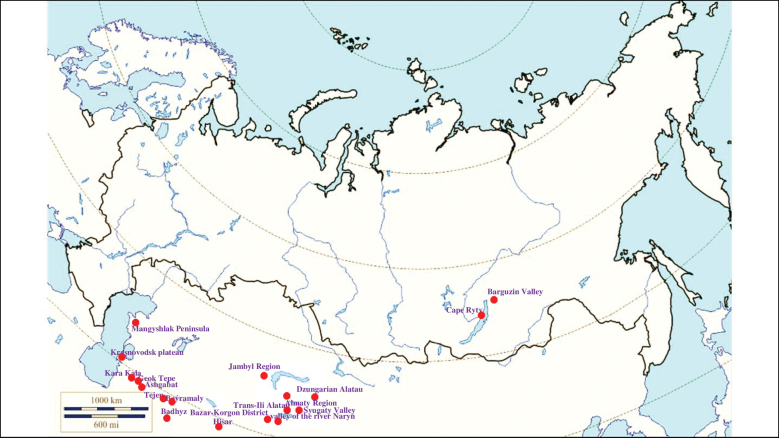
Map of Russia and neighboring countries showing the locations where *Ixodessubterraneus* was reported.

**(Fig. 29). Russia**: Transbikalia (Barguzin Valley, Cape Ryty) (Emelyanova 1972, as *I.arboricola*). **Kazakhstan**: Mangyshlak Peninsula (Maslennikova and Ushakova 1971), Jambyl Region (Kokuzek), Trans-Ili Alatau, Syugaty Valley (Maslennikova and Stogov 1974), Almaty Region (lower reaches of the Ili River) (Ushakova 1958, as *Ixodes* sp.), Dzungarian Alatau (Ushakova et al. 1976). **Kyrgyzstan**: Jalal-Abad Region (Bazar-Korgon District), the valley of the river Naryn (Grebenyuk 1966). **Turkmenistan**: Krasnovodsk plateau, outskirts of Geok Tepe, Kara Kala, Ashgabat, Tejen, Baýramaly, highland Badhyz (Filippova 1961; Kochkareva et al. 1971; Scherbinina 1973). **Tajikistan**: southern spurs of the Hisar Range – the vicinity of Hisar (Filippova 1977).

###### Ecology and other information.

*Ixodessubterraneus* is a parasite of birds nesting in ground burrows (Filippova 1977). The main part of its distribution lies in Kazakhstan and Middle Asia, the lesser part in Transbaikalia (Russia) (Filippova 1977). This tick species can be found in foothill dry steppes, as well as near and in deserts. This species was originally named *I.subterranus* in Filippova (1961) but amended to *I.subterraneus* in Filippova (1977).

#### ﻿Subgenus ﻿Scaphixodes Schulze, 1941: 491

##### ﻿ Ixodes berlesei

Taxon classificationAnimaliaIxodidaIxodidae

Birula, 1895

96428592-6011-5842-B903-917A0A88424D

Ixodes berlesei Birula, 1895: 353.

###### Recorded hosts.

**Aves**: *Apuspacificus* (Latham) (Pacific swift), *Corvusfrugilegus* Linnaeus (rook), *Falcorusticolus* Linnaeus (gyrfalcon), *Falcotinnunculus* Linnaeus (common kestrel), *Monticolasolitarius* (blue rock thrush), *Montifringillanivalis* (Linnaeus) (white-winged snowfinch), *Phoenicuruserythrogastrus* (Güldenstädt) (Güldenstädt’s redstart), *Phoenicurusochruros* (black redstart), *Phoenicuruserythronotus* (Eversmann’s redstart), *Plectrophenaxnivalis* (Linnaeus) (snow bunting), *Prunellacollaris* (Scopoli) (alpine accentor), *Sturnusvulgaris* (common starling), *Tichodromamuraria* (Linnaeus) (wallcreeper) (Filippova 1977; Voltsyt 1997).

###### Recorded locations

**Figure 30.**
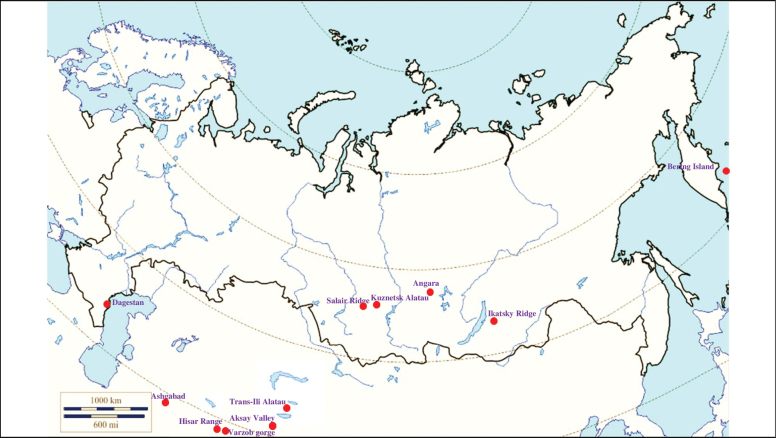
Map of Russia and neighboring countries showing the locations where *Ixodesberlesei* was reported.

**(Fig. 30). Russia**: Dagestan (Filippova 1977), Western Siberia – Salair Ridge, Kuznetsk Alatau (Chunihin 1967), Eastern Siberia – banks of the Angara River (Birula 1895) and Buryatia (Ikatsky Ridge) (Emelyanova et al. 1963), Bering Island (Voltsyt 1997). **Kazakhstan**: Trans-Ili Alatau (Grebenyuk 1966). **Kyrgyzstan**: Aksay Valley (Grebenyuk 1966). **Turkmenistan**: outskirts of Ashgabad (Filippova 1977). **Tajikistan**: Hisar Range, Varzob gorge (Ivanov 1945; Lotozky 1945).

###### Ecology and other information.

*Ixodesberlesei* is a little studied nidicolous tick occurring in the Greater Caucasus, as well as in Middle Asia and Siberia. There is one report about a finding of this tick on the Bering Island belonging to the Commander Islands in the Bering Sea, a female and three larvae collected 26 August 1990 from a snow bunting and deposited at the collection of the Zoological Museum of Moscow State University (Voltsyt 1997). The author states that the date of the tick collection indicates the presence of a permanent population of this species on the island because in the end of August birds usually already are prepared for the autumn migration after the breeding period, and, therefore, ticks could not have been transported there from the continent. Hence, we could assume that probably the real distribution of this tick is much wider and includes mountainous areas not only in a warmer and temperate climate but also in cooler tundra and other climatically similar landscapes. The snow bunting as a host of this species also was registered for the first time. Overall, its hosts include birds nesting usually in rocks and feeding on the ground and during the flight (Filippova 1977).

The type specimen is deposited at the Zoological Institute of the Russian Academy of Sciences - holotype: female; 683, [Russia, Siberia] Angara, 1867, Chekanovskii, type; AL I528. Description – Filippova 1977: 230–236 (female, nymph, larva; male unknown) (Filippova 2008).

##### ﻿ Ixodes caledonicus

Taxon classificationAnimaliaIxodidaIxodidae

Nuttall, 1910

0CA368A8-D9FB-5CD0-AB25-0A57FCDFE22A

Ixodes caledonicus Nuttall, 1910: Nuttall 1910: 408.
Ixodes caledonicus sculpturatus Schulze, 1929: 60; Arthur 1963: 53.
Ixodes gussevi Reznik, 1958: 457; Filippova and Panova 1975: 339.

###### Recorded hosts.

**Aves**: *Apuspacificus* (Pacific swift), *Corvuscorax* Linnaeus (common raven), *Corvuscornix* (hooded crow), *Columbalivia* (common pigeon), *Coloeusmonedula* (western jackdaw), *Falcoperegrinus* (peregrine falcon), *Monticolasolitarius* (blue rock thrush), *Oenantheoenanthe* (Northern wheatear), *Petroniapetronia* (rock sparrow), *Phoenicurus* sp. (redstart), *Tachymarptismelba* (Linnaeus) (Alpine swift) (Filippova 1977; Bolotin and Kolonin 1979).

###### Recorded locations

**Figure 31.**
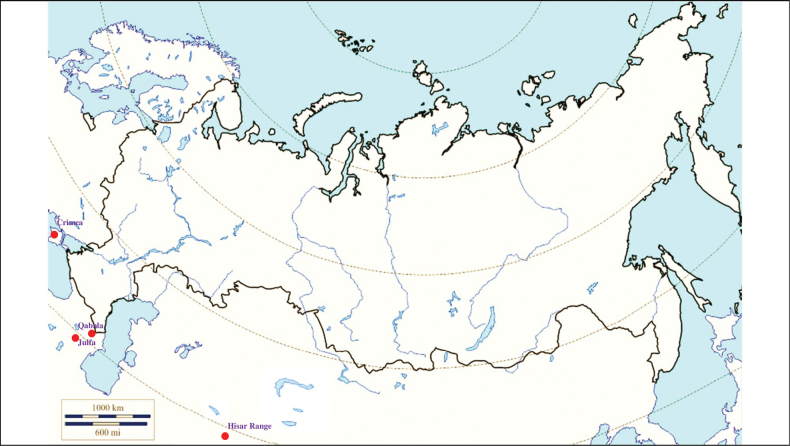
Map of Russia and neighboring countries showing the locations where *Ixodescaledonicus* was reported.

**(Fig. 31). Russia**: valley of the Zerkalnaya River (Bolotin and Kolonin 1979). **Ukraine**: Crimean Peninsula, in particular the Tarkhankut Peninsula and the cape Kazantyp (Emchuk 1960; Filippova 1977). **Azerbaijan**: Qabala (Reznik 1958), Julfa (Filippova and Panova 1975). **Tajikistan**: Hisar Range (Filippova and Panova 1975).

###### Ecology and other information.

*Ixodescaledonicus* is a little studied nidicolous tick species occurring in Europe as well as Western and Middle Asia. In Crimea this species is very rare and never has been found after 1980 (Nebogatkin 1998). Its hosts are birds that usually nest in rocks, feed on the ground, or feed and drink during flight (Filippova 1977).

##### ﻿ Ixodes semenovi

Taxon classificationAnimaliaIxodidaIxodidae

Olenev, 1929

3F7B082C-6426-53F4-B49A-A11C3468D8D0

Ixodes semenovi Olenev, 1929: 489.

###### Recorded hosts.

**Aves**: *Prunellacollaris* (alpine accentor), *Pyrrhocoraxpyrrhocorax* (Linnaeus) (red-billed chough) (Filippova 1977).

###### Recorded locations

**Figure 32.**
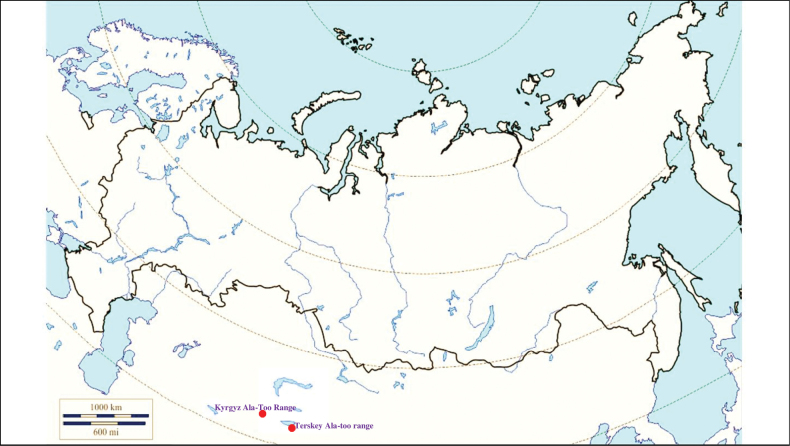
Map of Russia and neighboring countries showing the locations where *Ixodessemenovi* was reported.

**(Fig. 32). Kazakhstan**: Tian Shan – the northern slope of the Kyrgyz Ala-Too Range, the source of the river Merke (Jambyl Region) (Olenev 1929b). **Kyrgyzstan**: Terskey Ala-too Range (Grebenyuk 1961, 1966).

###### Ecology and other information.

*Ixodessemenovi* is a very rare species in the post-Soviet territories known only from Kazakhstan and Kyrgyzstan, from the Tian Shan, where it inhabits rocks at an altitude of 2000 m a. s. l. (Filippova 1977). The type specimen of *I.semenovi* is deposited at the Zoological Institute of the Russian Academy of Sciences: the holotype - female; Mi[ddle] Asia, Aleksandrovskii Mt. Range [Kirgizskii Ala Tau], source of Merke River, Aral-Tyube, from *Accentorcollaris*, 4.VII.1929, coll. I.A. Portenko; AL I549. Description – Filippova 1977: 219–223 (female, male, nymph; larva unknown) (Filippova 2008).

##### ﻿ Ixodes signatus

Taxon classificationAnimaliaIxodidaIxodidae

Birula, 1895

8FEA2EF0-E52A-580B-AF8B-F88E7CF4551E

Ixodes signatus Birula, 1895: 353.
Ixodes arcticus Osborn: Cooley and Kohls 1945: 201.
Ixodes parvirostris Neumann: Neumann 1904: 444.
Ixodes eudyptidis v. signata Birula: Neumann 1911: 21.

###### Recorded hosts.

**Aves**: *Phalacrocoraxcarbo* (Linnaeus) (great cormorant), *Urilepelagicus* (pelagic cormorant), *Urilepenicillatus* (Brandt) (Brandt’s cormorant), *Urileurile* (red-faced cormorant) (Filippova 1977).

###### Recorded locations

**Figure 33.**
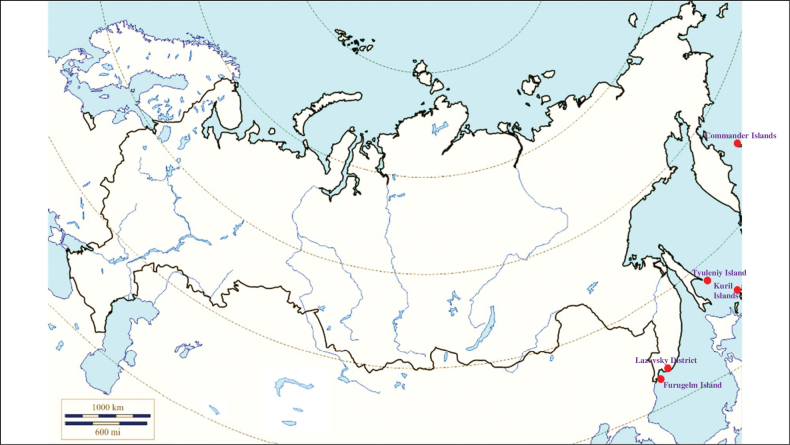
Map of Russia and neighboring countries showing the locations where *Ixodessignatus* was reported.

**(Fig. 33). Russia**: islands: Furugelm Island, Tyuleniy Island, the Kuril Islands – Paramushir, Urup and Makanrushi, the Commander Islands – the Kamen Ariy and the Bering Island (Kirschenblatt 1936; Pomerantsev 1950; Violovich 1958, 1962a; Leonova et al. 1971; Timofeeva et al. 1971, 1974; Lvov et al. 2014b); mainland – Primorsky Krai (Lazovsky District) (Kozlovskaya et al. 1968).

###### Ecology and other information.

*Ixodessignatus* is a nidicolous tick species occurring in several archipelagos and separate islands of the Russian Far East, as well as in Japan and the west coast of North America together with the Pacific islands nearby (Filippova 1977). It inhabits mostly coastal rocks being an obligate parasite of cormorants. Other birds, for example the Siberian thrush *Geokichlasibirica* (Pallas), are considered occasional hosts (Violovich 1962a). Findings in the mainland Eurasia are probably occasional cases of transportation (Kozlovskaya et al. 1968).

The type specimens of *I.signatus* are deposited at the Zoological Institute of the Russian Academy of Sciences (Filippova 2008) and include the lectotype: female; [Aleut Islands], Unalashka, 1846, coll. Voznesenskii, type; AL I358; paralectotypes: 8 females, 1 nymph, AL I358a; 2 females; CB I3170, I3171. Description – Filippova 1977: 204–210 (female, male, nymph, larva).

##### ﻿ Ixodes unicavatus

Taxon classificationAnimaliaIxodidaIxodidae

Neumann, 1908

070BB89B-9FF4-5546-ADC8-98E696B0A3F6

Ixodes unicavatus Neumann, 1908: 109; Schulze 1941: 491.
Ixodes tauricus Vshivkov & Filippova, 1957: 553; Gilot and Beaucournu 1973: 131.

###### Recorded hosts.

**Aves**: *Gulosusaristotelis* (European shag) (Linnaeus) (Filippova 1977).

###### Recorded locations

**Figure 34.**
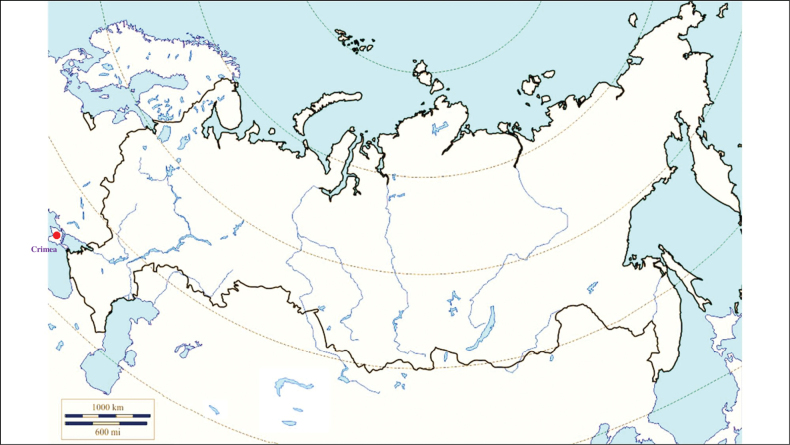
Map of Russia and neighboring countries showing the locations where *Ixodesunicavatus* was reported.

**(Fig. 34). Ukraine**: Crimean Peninsula, in particular the Tarkhankut Peninsula, The Kara Dag, the Kerch Peninsula, the cape Kazantyp (Emchuk 1960; Filippova 1977).

###### Ecology and other information.

*Ixodesunicavatus* is an endophilic tick occurring in Europe primarily in coastal areas of the Atlantic Ocean and which can be found in its hosts’ nests and under stones near them (Filippova 2007). It uses mostly cormorants - the European shag *Gulosusaristotelis* and the great cormorant *Phalacrocoraxcarbo* as hosts (Schulze 1932; Arthur 1963; Guiguen et al. 1987; Kolonin 2008). In Crimea, this species has been known from a little number of specimens (Serdjukova 1956; Emchuk 1960).

#### Subgenus ﻿﻿Trichotoixodes Reznik, 1961: 276.

##### ﻿ Ixodes brunneus

Taxon classificationAnimaliaIxodidaIxodidae

Koch, 1844

EED482A3-4C84-5819-93AD-1093548C1694

Ixodes brunneus Koch, 1844a: 232.
Ixodes californicus Banks, 1904: Keirans and Clifford 1978: 54.
Ixodes kelloggi Nuttall & Warburton, 1907: Cooley and Kohls 1945: 205.

###### Recorded hosts.

**Aves**: *Laniuscollurio* (red-backed shrike) (Filippova 1977)

###### Recorded locations

**Figure 35.**
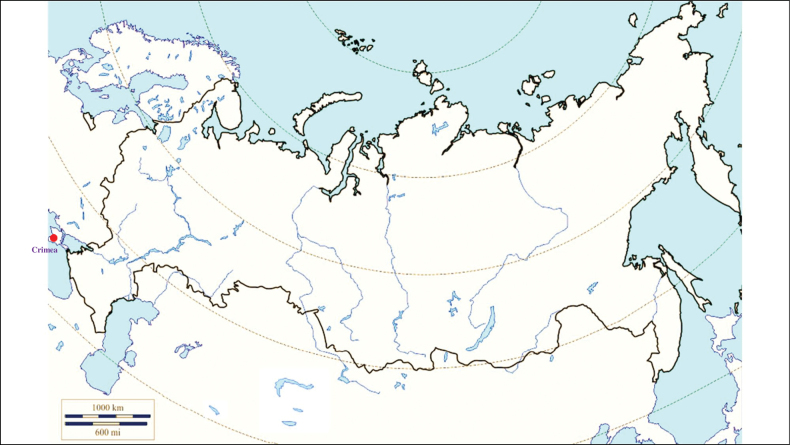
Map of Russia and neighboring countries showing the locations where *Ixodesbrunneus* was reported.

**(Fig. 35). Ukraine**: Crimea – Sudak City Municipality, the village Perevalivka (Filippova 1977).

###### Ecology and other information.

*Ixodesbrunneus* is a tick occurring mainly in the Americas being predominantly a parasite of passerine birds (Filippova 1977). The only record in Crimea on a red-backed shrike is considered a case of accidental introduction (Tsapko 2020).

##### ﻿ Ixodes frontalis

Taxon classificationAnimaliaIxodidaIxodidae

(Panzer, 1798)

32CBDE4F-6364-51FC-AE78-AF7CAE41D4F9

Acarus frontalis Panzer, 1798: 59, 23; Koch 1844a: 234.
Ixodes pallipes (Fabricius): Arthur 1963: 111.
Ixodes pari Leach, 1815: 399; Neumann 1911: 18.
Ixodes sturni Pagenstecher: Neumann 1901, 249.
Ixodes avisugus Berlese: Neumann 1899: 107.
Ixodes apronatus Kirshenblat, 1934: 257; Arthur 1963: 111.
Ixodes sigalasi Lamontellerie, 1954: 561; Lamontellerie, 1965: 87.

###### Recorded hosts.

**Aves**: *Alectorischukar* (chukar partridge), *Caprimulguseuropaeus* Linnaeus (European nightjar), *Chlorischloris* (European greenfinch), *Corvusfrugilegus* (rook), *Currucacommunis* (common whitethroat), *Currucacurruca* (lesser whitethroat), *Erithacusrubecula* (European robin), *Falcotinnunculus* (common kestrel), *Fringillacoelebs* (Eurasian chaffinch), *Fringillamontifringilla* (brambling), *Garrulusglandarius* (Eurasian jay), *Hippolaisicterina* (Vieillot) (icterine warbler), *Laniuscollurio* (red-backed shrike), *Luscinialuscinia* (thrush nightingale), *Lusciniamegarhynchos* (Brehm) (common nightingale), *Muscicapastriata* (spotted flycatcher), *Oenanthehispanica* (western black-eared wheatear), *Oenantheisabellina* (isabelline wheatear), *Parusmajor* (great tit), *Passerdomesticus* (house sparrow), *Passermontanus* (Eurasian tree sparrow), *Petroniapetronia* (rock sparrow), *Phoenicurusphoenicurus* (common redstart), *Phylloscopustrochilus* (willow warbler), *Phasianuscolchicus* (common pheasant), *Picapica* (Eurasian magpie), *Regulusregulus* (Linnaeus) (goldcrest), *Saxicolarubetra* (Linnaeus) (whinchat), *Streptopeliaturtur* (Linnaeus) (European turtle dove), *Turdusiliacus* (redwing), *Turdusmerula* (common blackbird), *Turdusphilomelos* (song thrush), *Turdustorquatus* Linnaeus (ring ouzel), *Turdusviscivorus* (mistle thrush) (Filippova 1977).

**Mammalia**: *Merioneslibycus* (Libyan jird) (Tsapko and Kotti 2017).

###### Recorded locations

**Figure 36.**
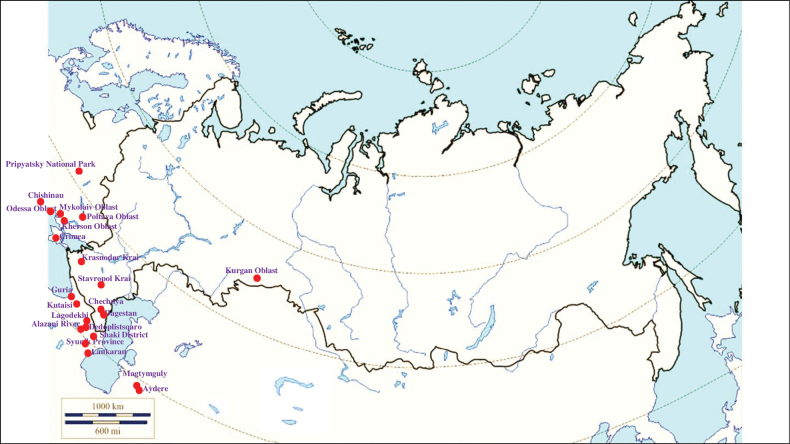
Map of Russia and neighboring countries showing the locations where *Ixodesfrontalis* was reported.

**(Fig. 36). Russia**: Kurgan Oblast – the rural locality Ketovo (Ruzsky 1929), Stavropol Krai (Reznik 1950; Guseva 1962; Tiflova et al. 1970), Krasnodar Krai; Chechnya (Marutyan 1963; Baisarova 2021), Dagestan (Gusev and Guseva 1960). **Ukraine**: Poltava Oblast (Olenev 1931a), Odesa Oblast, Mykolaiv Oblast, Kherson Oblast (Rusev 2009), Crimea (Filippova 1977). **Belarus**: Pripyatsky National Park (Tsvirko 2008). **Moldova**: Codru (Morozov et al. 2022), Olănești (Filippova 1977), Chishinau (Morozov and Proka 2012). **Armenia**: Syunik Province (former Goris Province) (Ogandzhanyan 1984). **Azerbaijan**: Alazani River (Ter-Vartanov et al. 1956), Lankaran (Filippova 1977), Shaki District (Tsapko and Kotti 2017). **Georgia**: Kutaisi, Lagodekhi, Dedoplistsqaro (Kirschenblatt 1936; Djaparidze 1960), Guria (Sukhiashvili et al. 2020). **Turkmenistam**: outskirts of Magtymguly, Aydere (Berdyev and Annaev 1997).

###### Ecology and other information.

*Ixodesfrontalis* is an exophilic tick species parasitizing primarily dendrophilic birds (Filippova 1977). It is relatively widely distributed throughout Europe, Western Asia, as well as North Africa (Filippova 1977; Estrada-Peña et al. 2018). *Ixodesfrontalis* is rare in most of its range. However, the place of mass reproduction of this species was discovered in Dagestan near the Sulak River in a big colony of rooks (Gusev and Guseva 1960). Under the nests in the rookery, a high, uncountable number of larvae of these ticks was observed. Often there were up to 5,000 individuals per m^2^ (Tsapko 2023).

Single collections of *I.frontalis* from mammals are known as exceptions. In Europe, adults were found on representatives of the mustelid family (Guglielmone et al. 2014). In the Shaki District of Azerbaijan (2 km north of the village of Şirinbulaq, 31 Oct 1956), two nymphs were taken from two Libyan jird *Merioneslibycus* (collections of R.B. Kosminsky and R.S. Karandina) (Tsapko and Kotti 2017). In addition, certain cases of attachments to humans are known (Gilot et al. 1997).

##### ﻿ Ixodes turdus

Taxon classificationAnimaliaIxodidaIxodidae

Nakatsudi, 1942

81A0E237-89BB-5934-8621-E51482423853

Ixodes turdus Nakatsudi, 1942: 287.

###### Recorded hosts.

**Aves**: *Turduspallidus* Gmelin (pale thrush) (Bolotin and Kolonin 1979).

###### Recorded locations

**Figure 37.**
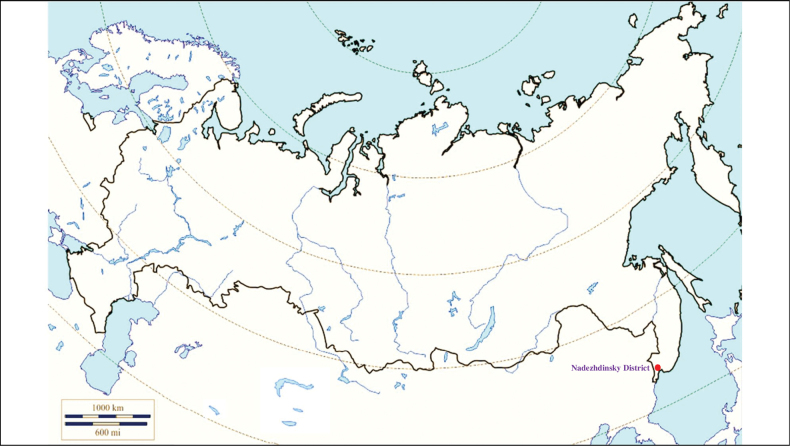
Map of Russia and neighboring countries showing the locations where *Ixodesturdus* was reported.

**(Fig. 37). Russia**: Primorsky Krai, Nadezhdinsky District, the right shore of the Razdolnaya River (Bolotin and Kolonin 1979).

###### Ecology and other information.

*Ixodesturdus* is a bird-associated tick species that can be found usually in East Asia, especially in Nepal, Korea, and Japan (Takahashi and Chunikhin 1972; Clifford et al. 1975; Ishiguro et al. 2000; Kim et al. 2009b; Sato et al. 2021). The single case of finding *I.turdus* in the Far East of Russia is considered a result of transportation (Bolotin and Kolonin 1979). Some occasions of parasitism on humans (Woo et al. 1990; Kadosaka and Hasegawa 1996), as well as on wild boars (Chae et al. 2017) are also recorded.

## ﻿Discussion

The territory of Russia and other post-Soviet countries reviewed here occupies a significant part of the Palearctic and its *Ixodes* tick fauna comprises in total approximately 37 species belonging to ten subgenera (Table 1). Some of these species are endemic. A significant ratio of these *Ixodes* species have a broad distribution area, as exemplified by *I.ricinus*, *I.persulcatus*, *I.trianguliceps*, *I.apronophorus*, *I.crenulatus*, *I.kaiseri*, *I.laguri*, *I.redikorzevi*, *I.eldaricus*, *I.frontalis*, and *I.lividus*. Moreover, the geographical range of some of these species also continues further to the west (into Europe) and to the south and east (to other parts of Asia).

**Table 1.** The list of *Ixodes* subgenera and species according to post-Soviet countries.

Tick subgenus	Tick species	Russia	Belarus	Ukraine	Moldova	Georgia	Armenia	Azerbaijan	Kazakhstan	Kyrgyzstan	Uzbekistan	Turkmenistan	Tajikistan
***Ceratixodes***	*I.uriae*	+											
***Eschatocephalus***	*I.simplex*	+		+				+					
	*I.vespertilionis*	+		+	+	+	+	+		+		+	+
***Filippoviella***	*I.ghilarovi*	+				+							
	*I.trianguliceps*	+	+	+	+	+	+	+					
***Ixodes***	*I.apronophorus*	+	+	+	+				+	+			
	*I.eldaricus*	+		+		+	+	+	+	+	+	+	+
	*I.kazakstani*								+	+			
	*I.kashmiricus*									+			
	*I.laguri*	+		+	+	+	+	+	+			+	
	*I.nipponensis*	+											
	*I.occultus*								+		+	+	
	*I.pavlovskyi*	+							+	+			
	*I.persulcatus*	+	+	+					+	+			
	*I.redikorzevi*	+		+	+	+	+	+	+	+	+	+	+
	*I.ricinus*	+	+	+	+	+	+	+	+			+	
	*I.sachalinensis*	+											
***Ixodiopsis***	*I.angustus*	+											
	*I.pomerantzevi*	+											
	*I.stromi*	+							+	+			+
***Monoixodes***	*I.maslovi*	+											
***Pholeoixodes***	*I.arboricola*	+	+	+	+	+	+	+		+			
	*I.cornutus*												+
	*I.crenulatus*	+	+	+	+	+	+		+	+	+	+	+
	*I.hexagonus*			+									
	*I.kaiseri*	+		+	+	+	+	+	+	+			
	*I.lividus*	+	+	+	+				+				
	*I.prokopjevi*	+											
	*I.subterraneus*	+							+	+		+	+
***Scaphixodes***	*I.signatus*	+											
	*I.berlesei*	+							+	+		+	+
	*I.caledonicus*	+		+				+					+
	*I.unicavatus*			+									
	*I.semenovi*								+	+			
***Trichotoixodes***	*I.brunneus* ^*^			+									
	*I.frontalis*	+	+	+	+	+	+	+				+	
	*I.turdus* ^*^	+											
**Total number of species**		**29**	**8**	**18**	**11**	**11**	**10**	**11**	**16**	**15**	**4**	**10**	**9**
**Total number of subgenera**		**9**	**4**	**6**	**5**	**5**	**5**	**6**	**4**	**5**	**2**	**5**	**5**

^*^ non-indigenous (transported) tick species in the reviewed territories.

Tick species like *I.ricinus* and *I.persulcatus* are able to live in a broad range of forest and forest-steppe biotopes and parasitize literally any vertebrate hosts among mammals, birds, and in some cases reptiles available in their habitats. Further species listed above parasitize species of those ecological groups of higher vertebrates which are widely distributed within the limits of the reviewed territories and even outside of them (like shrews, rodents, carnivores, and passerines), so this could explain the wide distribution of these species together with the presence of suitable hosts and biotopes. On the other hand, five tick species – *I.stromi*, *I.semenovi*, *I.signatus*, *I.uriae*, *I.occultus* – have more limited distribution areas, occurring only in certain habitats where they are specialized to parasitize an ecologically restricted range of hosts. There are at least five tick species (*I.angustus*, *I.pomerantzevi*, *I.nipponensis*, *I.kashmiricus*, *I.redikorzevi*) which have geographical ranges extending far beyond post-Soviet territories, and these also occur in neighboring and more distant countries sharing a similar fauna. The distribution areas of six further species (*I.berlesei*, *I.caledonicus*, *I.arboricola*, *I.subterraneus*, *I.simplex*, *I.vespertilionis*) cannot be defined more precisely, due to the limited number of their findings in locations distantly separated from each other. It is important to note here that these ticks are nidicolous parasites of birds and bats, therefore can be transported by their hosts to new habitats in other locations during migration, although it is not necessary that they will establish and form sustainable populations there. The distribution area of the tick *I.pavlovskyi* is also disjunct and populated by two different subspecies. Finally, there are four tick species known exclusively from the reviewed territories and certain locations by a very few records and their real distribution areas and biology are poorly studied, namely *I.cornutus*, *I.ghilarovi*, *I.maslovi*, and *I.prokopjevi*.

It is still questionable whether or not *I.brunneus* and *I.turdus* are indigenous in the examined geographical area. There have been no confirmations of stable populations of these two species in the locations where both species were found on migratory birds; both are known from these territories by single specimens outside their main distribution areas. Therefore, we suspect that these two tick species do not belong to the tick fauna of Russia and post-Soviet territories.

Among the reviewed *Ixodes* species, from the point of view of host preferences, there are both generalists and specialists. Rodents of the families Muridae and Cricetidae, as well as passerine birds, harbor the highest number of *Ixodes* species in the reviewed territories (Table 2). All these groups live almost everywhere in a great variety of biotopes, often in significant numbers, therefore playing an important role in diverse ecosystems and also having epidemiological significance as reservoirs of multiple tick-borne pathogens. Among the ticks in this review, 15 species parasitize murine rodents and 14 passerine birds (Table 2). Shrews (family Soricidae) also include a relatively high number of species which are ubiquitous and serve as typical hosts for certain *Ixodes* species, predominantly from the subgenera *Filippoviella* and *Ixodiopsis*.

**Table 2.** The list of *Ixodes* tick subgenera and species according to host taxa recorded in post-Soviet countries.

Tick subgenus	Tick species	Host taxonomic groups
***Ceratixodes***	*I.uriae*	**Aves**:
		Charadriiformes – Alcidae, Laridae;
		Suliformes – Phalacrocoracidae;
		Procellariiformes – Procellariidae
***Eschatocephalus***	*I.simplex*	**Mammalia**:
		Chiroptera
	*I.vespertilionis*	**Mammalia**:
		Chiroptera
***Filippoviella***	*I.ghilarovi*	**Mammalia**:
		Eulipotyphla – Soricidae;
		Rodentia – Cricetidae, Muridae
	*I.trianguliceps*	**Mammalia**:
		Eulipotyphla – Soricidae;
		Rodentia – Cricetidae, Muridae, Sminthidae
***Ixodes***	*I.apronophorus*	**Mammalia**:
		Eulipotyphla – Erinaceidae, Soricidae, Talpidae;
		Rodentia – Cricetidae, Muridae, Sciuridae, Sminthidae;
		Lagomorpha – Leporidae;
		Carnivora – Canidae, Mustelidae
	*I.eldaricus*	**Aves**:
		Galliformes;
		Passeriformes;
		Strigiformes
		**Mammalia**:
		Eulipotyphla – Soricidae;
		Rodentia – Cricetidae, Muridae
	*I.kazakstani*	**Aves**:
		Galliformes
		**Mammalia**:
		Lagomorpha – Leporidae;
		Rodentia – Cricetidae, Muridae, Gliridae
	*I.kashmiricus*	**Mammalia**:
		Rodentia – Muridae;
		Carnivora – Canidae;
		Artiodactyla – Bovidae
	*I.laguri*	**Mammalia**:
		Eulipotyphla – Erinaceidae;
		Rodentia – Cricetidae, Gliridae, Dipodidae, Muridae, Sciuridae, Spalacidae;
		Carnivora – Canidae, Mustelidae
	*I.nipponensis*	**Mammalia**:
		Rodentia – Cricetidae, Muridae
	*I.occultus*	**Mammalia**:
		Eulipotyphla – Soricidae;
		Rodentia – Cricetidae, Muridae, Sciuridae
	*I.pavlovskyi*	**Aves**:
		Anseriformes;
***Ixodes***	*I.pavlovskyi*	Columbiformes;
		Galliformes;
		Gruiformes;
		Passeriformes
		**Mammalia**:
		Eulipotyphla – Soricidae;
		Lagomorpha – Leporidae, Ochotonidae;
		Rodentia – Cricetidae, Muridae, Sciuridae, Sminthidae
	*I.persulcatus*	Any mammalian and avian hosts (rarely reptilian) available
	*I.redikorzevi*	**Aves**:
		Anseriformes;
		Columbiformes;
		Galliformes;
		Passeriformes;
		Pterocliformes
		**Mammalia**:
		Eulipotyphla – Erinaceidae, Soricidae;
		Lagomorpha – Leporidae;
		Rodentia – Cricetidae, Gliridae, Muridae, Sciuridae, Sminthidae, Spalacidae
		Carnivora – Canidae, Mustelidae
		**Reptilia**:
		Squamata – Lacertidae
	*I.ricinus*	**Mammalia, Aves, Reptilia**
	*I.sachalinensis*	**Mammalia**:
		Lagomorpha – Leporidae
***Ixodiopsis***	*I.angustus*	**Mammalia**:
		Eulipotyphla – Soricidae;
		Rodentia – Cricetidae, Muridae, Sciuridae, Sminthidae;
		Lagomorpha – Ochotonidae
	*I.pomerantzevi*	**Mammalia**:
		Eulipotyphla – Erinaceidae, Soricidae;
		Rodentia – Cricetidae, Muridae, Sciuridae
	*I.stromi*	**Mammalia**:
		Eulipotyphla – Soricidae;
		Rodentia – Cricetidae, Muridae;
		Lagomorpha – Ochotonidae;
		Carnivora – Mustelidae
***Monoixodes***	*I.maslovi*	Unknown
***Pholeoixodes***	*I.arboricola*	**Aves**:
		Accipitriformes;
		Bucerotiformes;
		Columbiformes;
		Passeriformes;
		Piciformes;
		Strigiformes;
		Falconiformes
	*I.cornutus*	**Mammalia**:
		Carnivora – Mustelidae
***Pholeoixodes***	*I.crenulatus*	**Mammalia**:
		Eulipotyphla – Erinaceidae;
		Rodentia – Cricetidae, Muridae, Sciuridae, Pálcaidé;
		Lagomorpha – Ochotonidae, Leporidae;
		Carnivora – Canidae, Felidae, Mustelidae, Procyonidae
	*I.hexagonus*	**Mammalia**:
		Eulipotyphla – Erinaceidae;
		Lagomorpha – Leporidae;
		Carnivora – Canidae, Felidae, Mustelidae
	*I.kaiseri*	**Mammalia**:
		Eulipotyphla – Erinaceidae;
		Rodentia – Hystricidae;
		Lagomorpha – Leporidae;
		Carnivora – Canidae, Felidae, Hyaenidae, Mustelidae
	*I.lividus*	**Aves**:
		Passeriformes;
		Coraciiformes
	*I.prokopjevi*	**Mammalia**:
		Eulipotyphla – Erinaceidae
	*I.subterraneus*	**Aves**:
		Galliformes;
		Passeriformes;
		Strigiformes;
		Falconiformes
***Scaphixodes***	*I.signatus*	**Aves**:
		Phalacrocoracidae – Phalacrocoracidae
	*I.berlesei*	**Aves**:
		Apodiformes;
		Passeriformes;
		Falconiformes
	*I.caledonicus*	**Aves**:
		Apodiformes;
		Columbiformes;
		Passeriformes;
		Falconiformes
	*I.unicavatus*	**Aves**:
		Suliformes – Phalacrocoracidae
	*I.semenovi*	**Aves**:
		Passeriformes
***Trichotoixodes***	*I.brunneus* ^*^	**Aves**:
		Passeriformes
	*I.frontalis*	**Aves**:
		Caprimulgiformes;
		Columbiformes;
		Galliformes;
		Passeriformes;
		Falconiformes
	*I.turdus* ^*^	**Aves**:
		Passeriformes

^*^ non-indigenous (transported) tick species in the reviewed territories.

In general, 18 *Ixodes* species are typically parasites of mammals from various taxonomic and ecological groups, 12 species prefer birds as hosts. Altogether, six species are generalists and therefore can parasitize virtually any available warm-blooded host species. All these species belong to the subgenus ﻿Ixodes. Ticks from other subgenera can attach to and feed from atypical hosts occasionally. Specific parasites of reptiles among *Ixodes* species are not known to occur in the reviewed territories, but some of the generalist species can parasitize these hosts, especially in the absence of their preferred ones. Sometimes even mass parasitism of *Ixodes* species can be observed on reptiles, as in the case of *I.redikorzevi*. Last, we can note that hosts of *I.maslovi* are still unknown, and the exact host range of *I.cornutus*, *I.ghilarovi*, *I.prokopjevi*, and *I.sachalinensis* also remains to be clarified.

## Supplementary Material

XML Treatment for Ixodes uriae

XML Treatment for Ixodes simplex

XML Treatment for Ixodes vespertilionis

XML Treatment for Ixodes ghilarovi

XML Treatment for Ixodes trianguliceps

XML Treatment for Ixodes apronophorus

XML Treatment for Ixodes eldaricus

XML Treatment for Ixodes kashmiricus

XML Treatment for Ixodes kazakstani

XML Treatment for Ixodes laguri

XML Treatment for Ixodes nipponensis

XML Treatment for Ixodes occultus

XML Treatment for Ixodes pavlovskyi

XML Treatment for Ixodes persulcatus

XML Treatment for Ixodes redikorzevi

XML Treatment for Ixodes ricinus

XML Treatment for Ixodes sachalinensis

XML Treatment for Ixodes angustus

XML Treatment for Ixodes pomerantzevi

XML Treatment for Ixodes stromi

XML Treatment for Ixodes maslovi

XML Treatment for Ixodes arboricola

XML Treatment for Ixodes cornutus

XML Treatment for Ixodes crenulatus

XML Treatment for Ixodes hexagonus

XML Treatment for Ixodes kaiseri

XML Treatment for Ixodes lividus

XML Treatment for Ixodes prokopjevi

XML Treatment for Ixodes subterraneus

XML Treatment for Ixodes berlesei

XML Treatment for Ixodes caledonicus

XML Treatment for Ixodes semenovi

XML Treatment for Ixodes signatus

XML Treatment for Ixodes unicavatus

XML Treatment for Ixodes brunneus

XML Treatment for Ixodes frontalis

XML Treatment for Ixodes turdus

## ﻿Additional information

### Author ORCIDs

Denis Fedorov https://orcid.org/0000-0003-0991-5728

Sándor Hornok https://orcid.org/0000-0002-1125-5178

### Data availability

All of the data that support the findings of this study are available in the main text.
